# Premutation in the Fragile X Mental Retardation 1 (*FMR1*) Gene Affects Maternal Zn-milk and Perinatal Brain Bioenergetics and Scaffolding

**DOI:** 10.3389/fnins.2016.00159

**Published:** 2016-04-19

**Authors:** Eleonora Napoli, Catherine Ross-Inta, Gyu Song, Sarah Wong, Randi Hagerman, Louise W. Gane, Jennifer T. Smilowitz, Flora Tassone, Cecilia Giulivi

**Affiliations:** ^1^Department of Molecular Biosciences, School of Veterinary Medicine Davis, CA, USA; ^2^Medical Investigations of Neurodevelopmental Disorders Institute, University of California, Davis Davis, CA, USA; ^3^Department of Pediatrics, University of California Davis Medical Center Sacramento, CA, USA; ^4^Department of Food Science and Technology and Foods for Health Institute, University of California, Davis Davis, CA, USA; ^5^Department of Biochemistry and Molecular Medicine, School of Medicine, University of California, Davis Davis, CA, USA

**Keywords:** bioenergetics, brain, *FMR1*, milk, mitochondria, premutation, Shank3, zinc

## Abstract

Fragile X premutation alleles have 55–200 CGG repeats in the 5′ UTR of the *FMR1* gene. Altered zinc (Zn) homeostasis has been reported in fibroblasts from >60 years old premutation carriers, in which Zn supplementation significantly restored Zn-dependent mitochondrial protein import/processing and function. Given that mitochondria play a critical role in synaptic transmission, brain function, and cognition, we tested FMRP protein expression, brain bioenergetics, and expression of the Zn-dependent synaptic scaffolding protein SH3 and multiple ankyrin repeat domains 3 (Shank3) in a knock-in (KI) premutation mouse model with 180 CGG repeats. Mitochondrial outcomes correlated with FMRP protein expression (but not *FMR1* gene expression) in KI mice and human fibroblasts from carriers of the pre- and full-mutation. Significant deficits in brain bioenergetics, Zn levels, and Shank3 protein expression were observed in the Zn-rich regions KI hippocampus and cerebellum at PND21, with some of these effects lasting into adulthood (PND210). A strong genotype × age interaction was observed for most of the outcomes tested in hippocampus and cerebellum, whereas in cortex, age played a major role. Given that the most significant effects were observed at the end of the lactation period, we hypothesized that KI milk might have a role at compounding the deleterious effects on the *FMR1* genetic background. A higher gene expression of *ZnT4* and *ZnT6*, Zn transporters abundant in brain and lactating mammary glands, was observed in the latter tissue of KI dams. A cross-fostering experiment allowed improving cortex bioenergetics in KI pups nursing on WT milk. Conversely, WT pups nursing on KI milk showed deficits in hippocampus and cerebellum bioenergetics. A highly significant milk type × genotype interaction was observed for all three-brain regions, being cortex the most influenced. Finally, lower milk-Zn levels were recorded in milk from lactating women carrying the premutation as well as other Zn-related outcomes (Zn-dependent alkaline phosphatase activity and lactose biosynthesis—whose limiting step is the Zn-dependent β-1,4-galactosyltransferase). In premutation carriers, altered Zn homeostasis, brain bioenergetics and Shank3 levels could be compounded by Zn-deficient milk, increasing the risk of developing emotional and neurological/cognitive problems and/or FXTAS later in life.

## Introduction

A 55–200 expanded CGG nucleotide repeats in the 5′-UTR of the fragile X mental retardation one gene (*FMR1*) constitutes the genetic hallmark of premutation carriers (OMIM#300623), whereas >200 repeats give rise to Fragile X syndrome (FXS; OMIM#300624), the leading inherited form of cognitive impairment (Kogan et al., 2008; Tassone et al., 2012; Battistella et al., 2013). Later in life, premutation carriers may have an increased risk of developing a neurodegenerative disorder known as Fragile X-associated tremor/ataxia syndrome [FXTAS (Kogan et al., 2008; Hagerman and Hagerman, 2013)]. Initially, this prevalent allelic variant was thought to be free of phenotypic traits; however, neurodevelopmental problems like autism spectrum disorder (ASD), attention deficit hyperactivity disorder (ADHD), anxiety, and other types of psychopathologies (Farzin et al., 2006; Tassone et al., 2012; Winarni et al., 2012; Wong et al., 2012; Battistella et al., 2013; Chonchaiya et al., 2013) have been reported in some young carriers, but not as often as in Fragile X syndrome [i.e., incidence of ASD in FXS is ~4-fold of that in premutation carriers (Farzin et al., 2006; Garcia-Nonell et al., 2008; Harris et al., 2008; D'Hulst et al., 2009; Zingerevich et al., 2009; Hagerman et al., 2010; Chonchaiya et al., 2013)].

Unlike the full mutation alleles, which undergo repeat-mediated gene silencing (Pieretti et al., 1991; Sutcliffe et al., 1992), premutation alleles are active and actually show normal or elevated *FMR1* mRNA levels (Tassone et al., 2000a,b, 2007). It is still not clearly understood how the premutation pathology arises and research to date has focused on how premutation alleles might trigger neurodegeneration through a gain-of-function (toxicity) RNA mechanism (Tassone et al., 2004; Hagerman and Hagerman, 2013). It has been suggested that the “excess” of premutation transcript may bind and sequester factors important for cell function (Sellier et al., 2010, 2013). Previous work from our laboratory conducted on fibroblasts from premutation individuals has shown that zinc (Zn) might also be included among these factors (Napoli et al., 2011). It has been shown that while *FMR1* gene expression could be high in some carriers, its product FMRP can be low (Tassone et al., 2000c; Tassone and Hagerman, 2003) which has been attributed to a reduced translation efficiency (Tassone and Hagerman, 2003). However, critical aspects of the pathology cannot be explained purely by an RNA-mediated process but rather to a protein-mediated neurodegeneration (Todd et al., 2013). Recently, the paradox of the RNA- mediated vs. protein-mediated toxicity in FXTAS, although still controversial (Banez-Coronel et al., 2012), has been partly explained by demonstrating that CGG repeats trigger repeat-associated non-AUG-initiated (RAN) translation (Todd et al., 2013) of a cryptic polyG-containing protein, FMRPolyG. Accumulation of FMRPolyG has been seen in ubiquitin-positive inclusions in *Drosophila*, mammalian cell cultures carrying the expansion, and brains of patients that died of FXTAS (Todd et al., 2013) and this lengthy polyglycine tract seems to be toxic to a number of cell types (Todd et al., 2013; Oh et al., 2015). So far, the relative contribution of RNA sequestration (of essential factors), low FMRP expression or RAN translation (of the toxic FMRPolyG) to the premutation pathology is unknown.

Adding to these mechanisms, bioenergetic deficits with increased oxidative stress biomarkers have been observed in post-mortem brain samples (Ross-Inta et al., 2010) and fibroblasts from premutation carriers (Ross-Inta et al., 2010; Napoli et al., 2011) and altered mitochondrial dynamics have been noted in neurons from a knock-in (KI) mouse model of *FMR1* premutation (Kaplan et al., 2012). These deficits seem to precede the occurrence of ubiquitin-positive intranuclear inclusions [considered a hallmark of FXTAS; (Greco et al., 2006)], and correlate with both CGG repeat expansion and severity of the phenotype (Ross-Inta et al., 2010; Napoli et al., 2011). Fibroblasts from >60 years old asymptomatic premutation carriers presented altered protein expression of the Zn transporters ZnT6/ZnT4 (Napoli et al., 2011) accompanied by mitochondrial dysfunction (MD; Ross-Inta et al., 2010; Napoli et al., 2011). This MD was mainly evidenced by an accumulation of precursor over mature mitochondrial proteins encoded by the nuclear DNA. This scenario was significantly reversed upon Zn supplementation, which allowed Zn-dependent import/processing pathways of nuclearly-encoded mitochondrial proteins to occur (Napoli et al., 2011).

Abnormal behavior (i.e., over-responsivity and hyperactivity-like behavior with acute Zn deficiency; ASD-like behavior secondary to prenatal Zn deficiency) was reported in young Zn-deficient mice with altered scaffolding elements within the postsynaptic density of excitatory synapses (Grabrucker et al., 2014). Indeed, it has been proposed that the ~50% incidence of Zn deficiency in children with ASD (Yasuda et al., 2011) has the potential to contribute to the etiology and/or morbidity of ASD via dysregulation of the synaptic Shank scaffolding (Grabrucker et al., 2014). Furthermore, some symptoms of ASD seem to lessen with Zn supplementation (Russo and Devito, 2011).

Collectively these studies suggest that the *FMR1* premutation affects Zn homeostasis, and that Zn deficits have a detrimental effect on behavior, opening the door for evaluating the effect of environmental stressors (such as Zn deficits) at compounding or initiating MD early in life and, possibly, predisposing young carriers to develop ASD, ADHD, and/or FXTAS at older age (Tassone et al., 2012; Winarni et al., 2012; Wong et al., 2012; Battistella et al., 2013).

Thus, we hypothesized that *FMR1* premutation would alter brain Zn homeostasis, bioenergetics, and protein expression of Zn-dependent scaffolding protein SH3 and multiple ankyrin repeat domains 3 (Shank3), outcomes which could be further affected by nursing on milk from premutation carriers. To test our hypothesis, *FMR1* gene expression, Fragile X mental retardation protein 1 (FMRP) protein expression, bioenergetics, Zn levels, and Shank3 protein expression were evaluated at post-natal days (PND) 0, 9 (or 7), 21, and 210 in cerebellum, hippocampus (both areas rich in Zn-containing neurons) as well as in cortex from a KI premutation mouse model. This murine model recapitulates some of the molecular, histological, and neurobehavioral deficits observed in premutation carriers (i.e., elevated *FMR1* mRNA, FMRP protein levels reduced or normal), and with age they develop intranuclear inclusions in neurons and astrocytes, ataxia-like mild motor dysfunctions, anxiety, and cognitive impairments (Hunsaker et al., 2009, 2011, 2012; Wenzel et al., 2010). To evaluate the effect of carriers' milk on the offspring's brain bioenergetics, a cross-fostering experiment was designed in which brain mitochondrial function was tested in suckling WT and KI pups at PND21. To complement the mouse model studies, Zn and Zn-associated outcomes were evaluated in breast milk from control and premutation nursing mothers.

Given the key role of Shank3 in post-synaptic neuron scaffolding and the contribution of mitochondria to the regulation of synaptic transmission, brain function, and cognition, we propose that early nutritional Zn interventions may represent a new preventive strategy in newborns of premutation mothers with the potential of lowering the risk of developing emotional or neurological symptoms later in life, emphasizing the interdependence between genetics and nutrition.

## Materials and methods

### Animals

This study was approved by the IACUC Committee at the University of California Davis, which is accredited by the American Association for the Accreditation of Laboratory Animal Care (IACUC-approved protocol number 17896). All animals were treated accordingly to the guidelines established by the NIH and the UCD animal welfare committee. Mice were monitored daily during the length of the experiments. Mice with signs of stress, weight loss >20%, paralysis, or any other serious disease, would have been euthanized immediately to avoid unnecessary pain or discomfort. However, none of the animals showed these signs of distress or were euthanized. No animal procedure was attempted without prior approval from the NIH and the UCD animal welfare committee, as well as all personnel was trained to handle animals under the current regulations. Wild-type female C57BL/6J mice were obtained commercially (Charles River, Wilmington, MA) and the knock-in (KI) mouse model of the premutation in the same genetic background (Wenzel et al., 2010) was from Dr. Robert Berman (University of California, Davis). The mice were housed in polycarbonate cages and fed *ad libitum*. Zn content of the diet used (Purina Pico chow 5058) was 1200 ppm, which with an average adult body weight of 30 g/mouse and a food intake of 2–5 g/mouse equals to 10–20 mg Zn/kg body weight *per diem*. Female mice during pregnancy increase their food intake by about two-fold and during lactation by about four-fold resulting in 20–40 mg Zn/kg body weight *per diem* during pregnancy and 40–60 mg Zn/kg body weight per day during lactation [reference values = 10 mg/kg and 30 mg/kg for adult and pregnant/lactating mice, respectively (Knapka et al., 1974; Luecke and Fraker, 1979; Beach et al., 1982)]. Mice were maintained on a 12 h light/dark cycle under controlled temperature and humidity. Mice were bred and allowed to deliver naturally. At PND0, 9 (or 7), 21, and 210, mitochondria were isolated from hippocampus, cerebellum and cortex from WT and KI male mice (*n* = 9–13 at each time point for a total of 54 WT and 55 KI). For the cross-fostering experiments (performed twice), at birth KI pups (males; *n* = 12; CGG repeats = 196 ± 6) and WT pups (males; *n* = 12; CGG repeats = 9.3 ± 0.2) were randomly foster-nursed either on KI dams (173 ± 4 CGG repeats) or on WT dams, with six pups on each dam. The KI pups were fed with the same frequency as WT animals. At PND21, WT, and KI dams were removed from pups for 2 h to control for effects of suckling on Zn transporter expression and localization, and subsequently were euthanized by CO_2_ asphyxiation. Mammary glands from lactating dams were removed and either snap-frozen in liquid nitrogen or stored in RNA later. At PND21 pups were also euthanized by CO_2_ asphyxiation. Euthanasia was performed by an experienced technician by way of inhalation of 30% CO_2_ (compressed gas cylinder). This method is considered acceptable by the 2000 Report of American Veterinary Medical Association (AVMA) Panel on Euthanasia and it has been approved by IACUC. The cortex, cerebellum, and hippocampus were removed post-mortem and processed immediately to isolate mitochondria.

### Isolation and purification of mitochondria from brain regions

Enriched mitochondrial fractions were obtained from cortex, cerebellum and hippocampus at PND0 (*n* = 13 WT and 13 KI), PND7 (*n* = 9 WT and 9 KI), PND21 (*n* = 12 WT and 12 KI), and PND210 (*n* = 11 WT and 12 KI). Intact, highly purified, non-synaptosomal mitochondria from the same brain tissues were isolated through a Percoll gradient as previously described in detail (Napoli et al., 2012, 2013), resuspended in iso-osmotic 150 mM KCl and immediately used for oxygen consumption measurements. Western blots to actin (cytosolic protein) and beta-ATPase (mitochondrial protein) showed a cytosolic contamination of the mitochondrial fraction of less than 2% [see (Napoli et al., 2013)].

### Primary neuronal cultures

To assess the contribution of glia to brain mitochondrial outcomes [as previously shown in Napoli et al. (2012)], isolated, intact primary neurons were obtained from WT and KI pups at PND0, using an established methodology (Brand, 1990; Jekabsons and Nicholls, 2004). Cultures of dissociated neurons were prepared as described in Kaplan et al. (2012) and kindly provided by Dr. Eitan Kaplan. Intact (non-permeabilized) neurons suspended in PBS supplemented with 10 mM glucose were used for evaluation of oxygen consumption followed by sequential additions of 5 μg/ml oligomycin and 20 μM FCCP (Napoli et al., 2011). The RCR under uncoupling conditions or RCRu in intact cells was calculated as the oxygen uptake ratio of State 3u (with FCCP) over that of oligomycin-induced State 4.

### Mitochondrial outcomes

Enriched mitochondrial fractions or in intact, purified mitochondria were used for evaluation of oxygen consumption using a Clark-type oxygen electrode (Hansatech, King's Lynn, UK) as described (Napoli et al., 2013). An aliquot (0.1–0.3 mg protein/ml) of mitochondria was added to the oxygen chamber in a buffer containing 0.22 M sucrose, 50 mM KCl, 1 mM EDTA, 10 mM KH_2_PO_4_, and 10 mM HEPES, pH 7.4. Oxygen consumption rates were evaluated in the presence of (i) 1 mM ADP plus 1 mM malate–10 mM glutamate followed by the addition of 5 μM rotenone; (ii) 10 mM succinate followed by the addition of 10 mM malonate; (iii) 10 mM α-glycerophosphate followed by addition of 3.6 μM antimycin A; and (iv) 10 mM ascorbate and 0.2 mM *N,N,N*′,*N*′-tetramethyl-*p*-phenylenediamine followed by the addition of 1 mM KCN. The activities of mitochondrial NADH oxidase, succinate oxidase, and cytochrome *c* oxidase were evaluated as the difference of oxygen uptake recorded before and after the addition of rotenone, malonate, antimycin A, and KCN respectively, and normalized by the activity of citrate synthase (a marker of mitochondrial mass). Citrate synthase activity was evaluated spectrophotometrically as described elsewhere (Napoli et al., 2013) using 1–2 μg of mitochondrial protein. The respiratory control ratio (RCR) with malate/glutamate as a substrate was calculated as the ratio between oxygen uptake rates in State 3 (with ADP or under phosphorylating conditions) and State 4 [rotenone-resistant oxygen uptake or non-phosphorylating conditions; (Napoli et al., 2013)].

### Western blots

For quantification of FMRP, Shank3, cytochrome *c* oxidase subunit IV (CCOIV), ATPase β-subunit (ATPB), β-actin, GAPDH, and tubulin expression levels, brain regions (hippocampus, cerebellum, and cortex) at PND9, 21, and 210 from WT and KI mice were homogenized in cold 20 mM HEPES, pH 7.4, added with protease and phosphatase inhibitor cocktails (Sigma, St. Louis, MO), and centrifuged at 13,000 × *g* for 10 min to eliminate particulate matter. A second set of Shank3 immunoblots (shown in Supplementary Figure 1) was carried out by using acetone precipitation to concentrate and partly delipidate the samples as previously described (Fujisawa et al., 2015). Thirty to fifty microgram of proteins were solubilized in SDS sample buffer (Life Technologies, Grand Island, NY) and loaded onto a 4–12% bis-tris gel (Life Technologies) as previously described (Napoli et al., 2013). After transferring proteins with an iBlot apparatus (Life Technologies), membranes were blocked with LI-COR blocking buffer (LI-COR Biosciences, Lincoln, NE) for 1 h at room temperature and subsequently probed with anti-FMRP antibody (Sigma, St. Louis, MO; 1:700 dilution), anti-Shank3 antibody (Santa Cruz Biotechnologies, Dallas, TX; 1:500 dilution), anti-CCOIV antibody (Cell Signaling, Danvers, MA, 1:1000 dilution), anti ATPB (BD Biosciences, San Jose, CA, 1:3000 dilution) overnight at 4°C, and with anti-β-actin (Sigma, St. Louis, MO; 1:20,000 dilution) for 1 hour at room temperature. Tubulin (Proteintech, Chicago, IL; 1:1,000 dilution) and GAPDH (Santa Cruz Biotechnology, Dallas, TX, 1:300 dilution) were used as loading controls. Secondary antibodies were from LI-COR (Lincoln, NE; 1:10,000 dilution). Membranes were visualized with the use of the Odyssey Infrared Imaging System (LI-COR) and densitometry analysis carried out with the Carestream software (Napoli et al., 2013).

### Gene expression of *ZnT4*/*T6* in mammary glands from lactating WT and KI dams

RT-qPCR performed with three different sets of commercially available primers from Life Technologies showed low specificity and efficiency. Thus, the gene expression of *ZnT4* and *ZnT6* was assessed by PCR. Total RNA was isolated from homogenized mammary gland following the manufacturer's instructions (Qiagen). Concentration and purity of RNA was measured at an absorbance of 260 nm and 280 nm using the Tecan i-control 1.6 software (v.1.6.19.2) on Tecan infinite M200 Nanoquant (Tecan, Austria). Following RNA isolation, 2 μg of the RNA was used to make cDNA with the Qiagen Quantitech RT Kit following the manufacturer's instructions. Following cDNA synthesis, *ZnT4*, and *ZnT6* gene expression was performed by Touchdown PCR in a 96-well plate in a 25 μl reaction volume containing: 100 ng of cDNA, 2.5 μl of 10x Advantage 2 PCR buffer (Clontech, Mountain View, CA), 0.5 μl of 50X Advantage 2 Polymerase Mix (Clontech), 1 μl of 10 mM dNTPs (Life Technologies, Grand Island, NY), and 0.5 μl of each 10 mM forward and reverse primer. Mouse primers for *ZnT4* were: forward 5′-CTCCAGGCCGACGATGACT-3′; reverse 5′-GTGTCCACTAGATACCATGCTTGG-3′. Primers for *ZnT6* were: forward 5′-CAGACCTTAGCCGCAGCTTG-3′; reverse 5′-GGTCTGAGAAGTTTAGGACGTTGG-3′. PCR cycling parameters were: 95°C for 3 min; 10 cycles of: 94°C for 15 s, 65°C for 30 s (with 1°C decrease at each cycle), and 72°C for 40 s; 30 cycles of: 95°C for 15 s, 55°C for 30 s, and 72°C for 40 s; 5 min at 72°C, 15°C hold step. PCR was performed on a Mastercycler EP Realplex thermocycler (Eppendorf, Westbury, NY). PCR products were separated in a 1.3% agarose gel in the presence of ethidium bromide, and the fragments had the expected sizes of 585 bp (*ZnT4*) and 537 bp (*ZnT6*). Gene expression of *ZnT4* and *ZnT6* was normalized by that of *GAPDH*. Expression of *GAPDH* was performed in a 96-well PCR plate by Real Time RT PCR with 100 ng of cDNA, TaqMan Universal PCR Mastermix (Life Technologies, Grand Island, NY), and 0.5x *GAPDH* primer-probe mix (Life Technologies, Grand Island, NY). Amplification was performed using the default cycling parameters of 2 min at 50°C, 10 min at 95°C, and 40 cycles of 15 s at 95°C, and 60 s at 60°C. PCR product was separated in a 3% agarose gel electrophoresis in the presence of ethidium bromide with an expected fragment size of 107 bp.

### Human subjects and breast milk samples

Control breast milk samples were obtained from 25 healthy women who gave birth to healthy term infants enrolled in the UC Davis Foods for Health Institute Lactation Study (ClinicalTrials.gov Identifier: NCT01817127). Donors filled out detailed health history questionnaires regarding demographics, anthropometrics, pregnancy history, current, and prior health history, current dietary intake habits and restrictions, physical activity level, as well as medication and supplementation intake history during the third trimester of pregnancy and 1 year postpartum. Half of the control donors reported intake of prenatal vitamins and minerals during the post-partum period. Breast milk was also obtained from one control and five premutation lactating mothers seen through the Fragile X Treatment and Research Center at the M.I.N.D. Institute at the University of California Davis Medical Center. All milk samples (5–30 ml) were obtained between 8 and 20 lactation weeks. These women delivered their infants between 38 and 41 weeks of gestation. Fresh milk samples were collected using hand expression at the conclusion of a morning breastfeed (between 09.00 and 12.00 h). All milk samples were frozen within 1 h of expression at –20°C, and transferred to –80°C until analyzed. Genotyping for these individuals was performed by extracting gDNA from milk samples and following the procedure described before (Tassone et al., 2012). The UC Davis Institutional Review Board approved all aspects of the study (IRB # 200917212-1) and informed consent was obtained from all donors.

### Activity of milk alkaline phosphatase

ALP activity was measured in breast milk and the method optimized using unpasteurized cow's milk (from Trader Joe's, Davis, CA). ALP activity was evaluated in 6 μl (correspondent to ~12–13 μg of protein) of breast milk samples using the QuantiChrom Alkaline Phosphatase Assay Kit (BioAssay Systems, Hayward, CA). The assay utilizes *p*-nitrophenyl phosphate that is hydrolyzed by ALP into a color product, which is measured at λ = 405 nm for 5 min at 37°C. Total protein concentration of breast milk was measured with the Pierce BCA Protein Assay kit (Thermo Scientific, Waltham, MA). ALP activity was expressed either as μmol × (min × l milk)^−1^ or nmol × (min × mg protein)^−1^. The detection limit of this assay was (mean ± SEM) 0.050 ± 0.001μmol × (min × l)^−1^ (*n* = 16) and the inter-assay CV was 8.1% (*n* = 32).

### Milk lactose determination

Lactose was determined by an enzymatic spectroscopic method according to the manufacturer's instructions (Biovision, Milpitas, CA). The recovery of a known amount of lactose added to the milk samples was (mean ± SEM) 101 ± 0.9 % (*n* = 12). The detection limit of this assay was 0.025 ± 0.002 nmol galactose (*n* = 12) and the inter-assay CV was 10.2% (*n* = 42).

### Determination of Zn levels in murine brain and human milk

Measurements of Zn in murine brain regions and human milk were carried out with the Zn fluorophore zinquin (Sigma-Aldrich, St. Louis, MO) essentially as previously described (Zalewski et al., 2006) with some modifications. To evaluate total Zn levels in mouse brain regions, homogenized samples for Western blot analysis were used. One-hundred microgram of brain protein was added to each well of a 96-well-microplate to a final volume of 100 μl in the presence of 10 μM zinquin dissolved in Zn-free Hank's balanced salt solution (HBSS, in mM: 0.03 Na_2_HPO_4_, 0.4 KH_2_PO_4_, 4.2 NaHCO_3_, 5.4 KCl, 5.6 D-glucose, 137 NaCl; pH 7.4) following the addition of Zn-free ovalbumin to a final concentration of 0.3 mg/ml (to prevent precipitation of the lipophilic zinquin from aqueous solutions). Samples were incubated in the dark for 40 min at 22°C, and fluorescence was evaluated at excitation and emission wavelengths of 365 and 510 nm, respectively.

For the determination of Zn in milk, 5 μl of milk were used and the assay carried out essentially as described for brain samples, but with and without 10 mM EGTA. EGTA-containing samples were used to obtain the non-labile Zn fluorescence, as EGTA removes any free Zn-related fluorescence. Free Zn concentrations were evaluated by subtracting the fluorescence in the presence of EGTA by the total fluorescence without EGTA, and converting fluorescence values into Zn concentrations using the linear part of a calibration curve performed with 1 to 25 μM ZnSO_4_. To confirm that milk or brain samples did not display any intrinsic fluorescence at the excitation and emission wavelengths used, blanks without zinquin were prepared. No unspecific fluorescence was recorded in any of the samples in the absence of zinquin.

### Human fibroblasts collection and outcomes

Skin biopsies from controls, premutation and full mutation carriers were obtained from subjects recruited through the Fragile X Treatment and Research Center at the M.I.N.D. at the University of California Davis Medical Center. The UC Davis Institutional Review Board approved all aspects of the study and informed consent was obtained from the parents of the children. All fibroblasts were grown in Minimum Essential Medium (MEM) supplemented with 15% FBS, 2 mM glutamine and 1 mM sodium pyruvate. FMRP levels were evaluated by Western blots upon lysis of whole cells in RIPA (25 mM MOPS, 150 mM NaCl, 1 mM EDTA, 1% Triton, 0.1% SDS, and 1% DOC, pH 7.5), and centrifugation at 12,000 × *g* for 10 min. For *FMR1* gene expression, RNA was isolated from 10^6^ fibroblasts using the RNeasy Plus Mini Kit from Qiagen (cat. no. 74134) following the manufacturer's protocol. cDNA was synthesized from the RNA using Qiagen's Quantitect RT kit following manufacturers recommendations. RNA and cDNA concentrations were determined using the Tecan Infinite M200 Nanoquant plate reader (Tecan, Austria). Primer/probe mix was purchased from Life Technologies (Grand Island, NY, USA) for *FMR1* and *XRCC5* (housekeeping gene) were also obtained by Life Technologies. Sequences of commercial primers and probes are proprietary. cDNA was diluted to 5 ng/μl and served as stock template for QRTPCR. QRTPCR was performed in a Mastercycler EP Realplex thermocycler (Eppendorf, Westbury, NY). Amplification was performed using the following cycling parameters: 2 min at 50°C, 95°C for 10 min, followed by 40 cycles of 15 s at 95°C and 1 min of at 60°C. The mean cycle time was obtained by double derivatives (CalqPlex algorithm; Eppendorf, Westbury, NY) and designated as Ct. Each sample was analyzed in triplicates, and positive and negative controls were run on each plate. Coefficient of variation (CV) was 0.3% or less on average. The gene expression of *FMR1* was determined by the comparative Ct method using the following equation: 2ΔCt, where ΔCt = Ct_Target_–Ct_Housekeeper_. The gene expression fold change (premutation or full muation/Control) was determined using the ΔΔCt method using the following equation: 2^(ΔCtFXTAS−ΔCtControl)^. Fold changes were calculated based on their age matched controls. Values were converted to positive or negative values to indicate up or down gene regulation. Coupling between electron transport and ATP production were tested in whole, intact cells as previously described (Napoli et al., 2011).

### Statistical analysis

Data are expressed as mean ± SEM. Statistical analysis was performed either by Student's *t*-test (for comparisons between WT and KI), or by Two-way ANOVA, followed by the Tukey's HSD *post-hoc* test for multiple comparisons with the GraphPad Prism 6.0 software (San Diego, CA). Significance was set at *p* ≤ 0.05. Unless otherwise noted, experiments were run in triplicates (technical replicates) and performed at least in 2–3 separate occasions (biological replicates). To determine the minimum number of mice/group, we used an a priori G test (software STATsimple; v. 2.0.5) with selected mitochondrial outcomes that indicated 4 mice as optimal given an alpha of 0.05 with an actual power of 0.993. Considering the yield of mitochondria from mouse brain (a limiting step), sample needed for assay in triplicates, and the assay parameter (polarography) that requires the largest amount of sample, 5 mice/group were used at a minimum for each time point, unless otherwise stated.

## Results

### Decreased FMRP protein levels in brain from young KI mice

**Figure 1:**
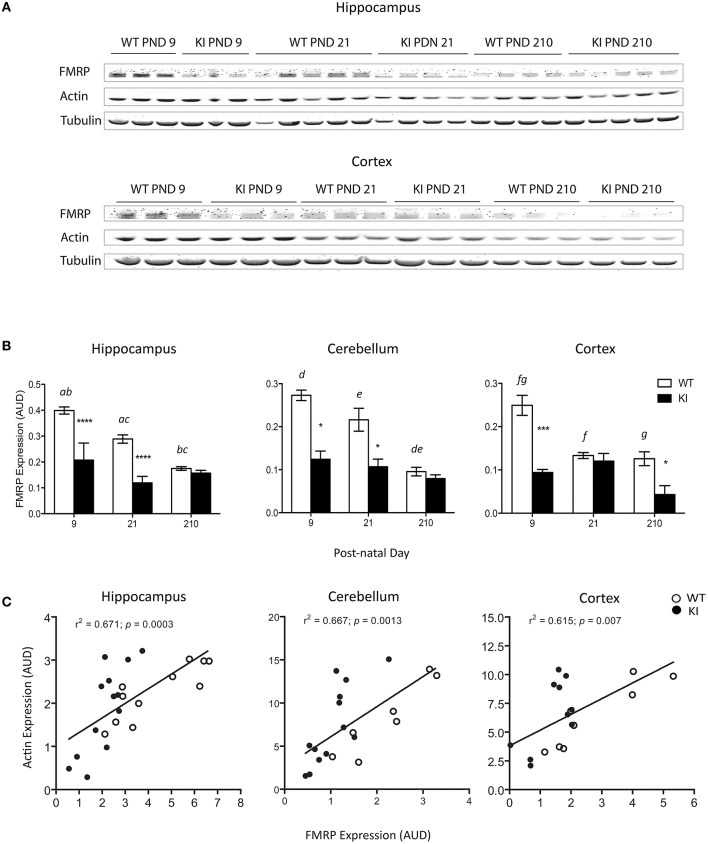
**Changes in FMRP protein expression in brain from WT and KI mice. (A)** Representative Western blots of FMRP and actin protein expression levels in hippocampus and cortex of WT and KI mice. Tubulin was used as loading control. FMRP protein levels of KI mice hippocampus and cerebellum were 37 and 45% of WT at post-natal day (PND) 9, and 41 and 50% of WT at post-natal day 21. In cortex, FMRP protein levels in KI mice were 38% of WT at post-natal day 9. **(B)** Time-dependent changes in FMRP protein levels in hippocampus, cerebellum, and cortex respectively. Data are reported as mean ± SEM, *n* = 3–5 per genotype per time point. Statistical analysis was performed by Two-way ANOVA. *Post-hoc* analysis performed by Tukey's HSD test revealed significant differences between WT and KI, indicated in the figure by asterisk as follows. Hippocampus: ^****^p < 0.0001; Cerebellum: ^*^p = 0.0110; Cortex: ^**^p = 0.0003, ^*^p = 0.0363, ^***^p = 0.0001. Statistically significant differences among time points are indicated by letters with the following *p* values. *p* = 0.0029 (*a*), *p* < 0.0001 (*b*), *p* = 0.0009 (*c*), *p* = 0.0260 (*d*), *p* = 0.0101 (*e*), *p* = 0.0033 (*f*), *p* = 0.0020 (*g*). For more statistical details on the genotype, age, and genotype × age effect see Table 3. **(C)** Correlation between FMRP and actin in the same brain areas. AUD, Arbitrary Units of Densitometry.

Male, hemizygous KI mice were used as a murine model of the *FMR1* premutation [CGG repeat expansion = 196 ± 6; (Wenzel et al., 2010)]. This model recapitulates most deficits observed in premutation carriers (Hunsaker et al., 2009, 2011, 2012; Wenzel et al., 2010). Despite the relatively high CGG expansions, the expression of the *FMR1* gene product FMRP, was still detectable at PND9, PND21, and PND210 in hippocampus, cerebellum, and cortex and not absent as observed in models of Fragile X syndrome [(Zalfa et al., 2003); Figures 1A,B]. Consistent with other reports (Ludwig et al., 2014), FMRP protein expression decreased with age in all brain areas in WT (Figure 1B); conversely, no age-dependent effect was observed on FMRP expression of KI mice in any of the brain regions tested. At PND9, 40-50% of FMRP control values were observed in hippocampus, cerebellum, and cortex of KI mice, effect that lasted into PND21 for the first two tissues (Figure 1B).

Interestingly, in all three-brain regions the expression levels of FMRP were followed by those of β-actin (Figure 1C). The correlation between actin and FMRP protein levels is consistent with the role of FMRP as regulator of actin filaments organization and dynamics (Castets et al., 2005; Nolze et al., 2013), a key process in the morphogenesis of dendritic spines (Castets et al., 2005), influencing the effect of actin on mitochondria distribution and function (Kusano et al., 2000; Xu et al., 2001; Dugina et al., 2009), mitochondrial fission (Korobova et al., 2013; Hatch et al., 2014; Li et al., 2015), short-distance mitochondrial movements (Boldogh and Pon, 2006), mitochondria quality control (Higuchi et al., 2013), mitochondria clustering and reactive oxygen species (ROS) generation (Li et al., 2004).

### Deficits in bioenergetics in brain regions from young KI mice

Different segments of the electron transport chain from brain mitochondria (hippocampus, cerebellum, and cortex) were tested for their capacity to generate ATP in WT and KI mice: NADH oxidase (using NAD-linked substrates such as glucose and comprising Complex I, III, IV, and V), succinate oxidase (using FAD-linked substrates such as fatty acids and comprising Complex II, III, IV, and V), as well as citrate synthase activity as a normalizing marker of mitochondrial mass. Complex IV, or cytochrome *c* oxidase (CCO), activity, and coupling between ATP synthesis and electron transfer (as judged by the RCR) were also tested.

**Figure 2:**
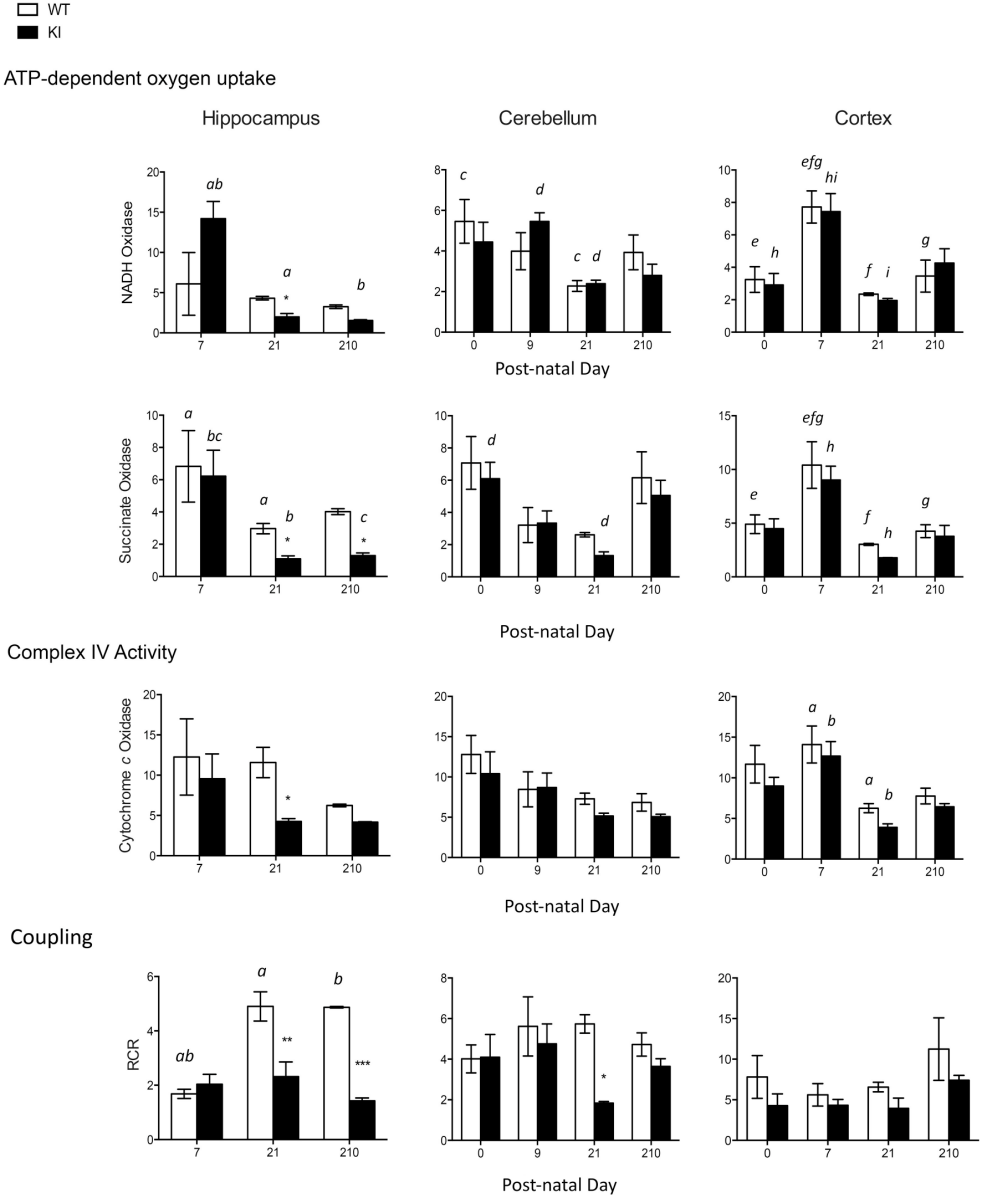
**Brain bioenergetics of KI mice during neurodevelopment and adulthood**. Mitochondria were isolated from cortex, cerebellum, and hippocampus of WT and KI pups as described in the Methods section. Activities of NADH oxidase, succinate oxidase, and cytochrome *c* oxidase (CCO), and respiratory control ratio (RCR) were evaluated at PND0 (cerebellum and cortex only, due to the scarcity of hippocampal tissue), PND7, PND21, and PND210. Data are reported as mean ± SEM, *n* = 3–7 per genotype per time point. Statistical analysis was performed by Two-way ANOVA. *Post-hoc* analysis performed by Tukey's HSD test revealed significant differences between WT and KI indicated by asterisks as follows NADH oxidase: ^*^p = 0.0468; Succinate oxidase: ^*^p = 0.0466 at PND21, ^*^p = 0.0423 at PND210; Cytochrome *c* oxidase: ^*^p = 0.0484; Coupling: ^**^p = 0.001, ^***^p = 0.0004, ^*^p = 0.0496. Letters indicate statistically significant differences amongst time points as follows. NADH oxidase: *p* = 0.0291 (*a*), *p* = 0.0033 (*b*), *p* = 0.0166 (*c*), *p* = 0.0320 (*d*), *p* = 0.0036 (*e*), *p* = 0.0001 (*f*), *p* = 0.0113 (*g*), *p* = 0.0032 (*h*), *p* = 0.0004 (*i*); Succinate oxidase: *p* < 0.0001 (*a*), *p* = 0.0002 (*b*), *p* = 0.0240 (*c*), *p* = 0.0434 (*d*), *p* = 0.0283 (*e*), *p* = 0.0009 (*f*), *p* = 0.0250 (*g*), *p* = 0.0013 (*h*); Cytochrome *c* oxidase: *p* = 0.0053 (*a*), *p* = 0.0035 (*b*). Coupling: *p* = 0.0003 (*a*), *p* = 0.0010 (*b*). Further statistical details on the genotype, age, and genotype × age effect can be found in the legend of Table 3.

At PND0-7, no statistically significant differences were observed in mitochondrial outcomes between WT and KI pups in any of the brain regions tested (Figure 2). At PND21, hippocampus was the most affected brain area in KI pups, with significant decreases in NADH oxidase, succinate oxidase, and CCO activities, as well as increased uncoupling between ATP production and electron transfer, relative to WT (Figure 2). At PND21, uncoupling was also evident in cerebellum of KI pups, but to a lesser extent than hippocampus (Figure 2), whereas in adult age (PND210) succinate oxidase, and coupling (hippocampus) were still significantly lower in KI hippocampus than age-matched WT mice. No difference was observed in any of the outcomes in cortex at any of the time points (Figure 2).

**Figure 3:**
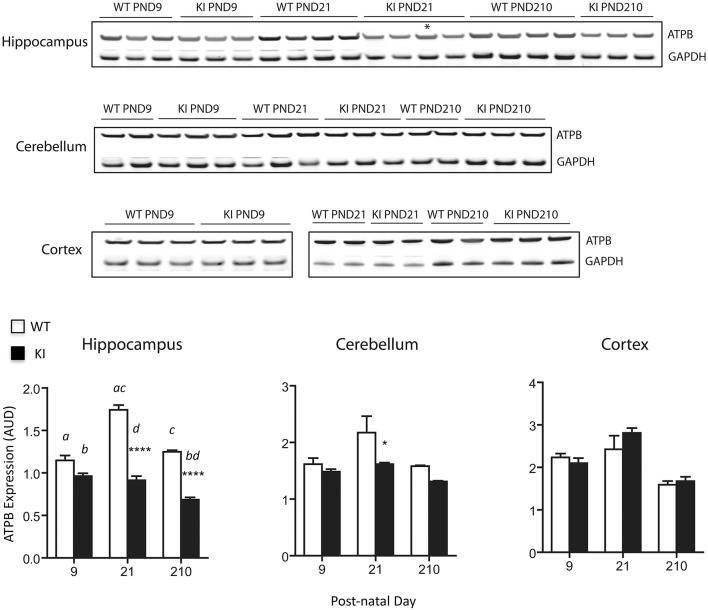
**Protein expression levels of mitochondrial ATPase β-subunit in brain from KI mice**. ATPase β-subunit protein expression (normalized to GAPDH) was evaluated at PND9, 21 and 210 in hippocampus, cerebellum, and cortex lysates. Asterisk in the immunoblot image of hippocampus denotes a 90 CGG KI sample. Densitometry data for this sample have not been taken into account for averages calculation of KI. Cortex samples were ran in two different gels (PND9 and PND21-210) and are shown separately. Data are reported as mean ± SEM, *n* = 3–5 per genotype per time point, ran in triplicates. Statistical analysis was performed by Two-way ANOVA. *Post-hoc* analysis was performed by Tukey's HSD test for multiple comparisons. Significant difference between WT and KI are indicated by asterisks as follows. ^****^p < 0.0001; ^*^p < 0.0492. Statistically significant differences among time points are indicated by letters as follows: *p* < 0.0001 (*a, c*), *p* = 0.0087 (*b*), *p* = 0.0222 (*d*). Further, statistical details on the genotype, age, and genotype × age effect can be found in Table 3. AUD, Arbitrary Units of Densitometry.

Mitochondrial protein levels (relative to total protein) were evaluated by measuring the expression of the abundant protein ATPase β-subunit of Complex V [ATPB; (Elfering et al., 2004; Haynes et al., 2010), Figure 3]. Lower levels of ATPB were noted at PND21 and lasting into adulthood (PND210) in hippocampus of KI mice, while in cerebellum significant ATPB deficits were observed only at PND21 (Figure 3). No statistically significant differences were observed in cortex at any time point (Figure 3).

Taken together, these results point toward a generalized OXPHOS deficit in premutation pups' hippocampus, characterized by lower ATP production with both NAD- and FAD-linked substrates, accompanied by lower expression of ATPB. These deficits were less evident in cerebellum (uncoupling and lower ATPB expression were evident at PND21 only), with no apparent involvement of cortex.

The OXPHOS changes observed in brain mitochondria could not be ascribed to a specific subcellular localization (e.g., synaptosomal mitochondria derived only from the termini of neurons vs. free, non-synaptosomal mitochondria) because the majority of brain mitochondria [88% of all mitochondria (Rendon and Masmoudi, 1985)] are non-synaptosomal (Napoli et al., 2012). As such, no OXPHOS differences were observed between mitochondria-enriched fractions and highly purified, non-synaptosomal mitochondria at PND21 in any brain region from WT and KI (Table 1).

**Table 1:** **Comparison of outcomes in mitochondria-enriched fractions vs. purified, non-synaptosomal mitochondria from cerebellum and cortex at PND21 in WT and KI mice**.

**Outcomes**	**Cerebellum**				**Cortex**			
	**Total mitochondria**		**Non-synaptosomal mitochondria**		**Total mitochondria**		**Non-synaptosomal mitochondria**	
	**WT**	**KI**	**WT**	**KI**	**WT**	**KI**	**WT**	**KI**
**OXYGEN UPTAKE RATES**								
NADH oxidase	5.38 ± 0.01	4.5 ± 0.3	52.4 ± 1.3	58.3 ± 5.6	8.8 ± 3.3	8.4 ± 1.0	50 ± 11	45 ± 6
Succinate oxidase	6.31 ± 0.02	2.33 ± 0.01^**^	47.1 ± 1.0	26 ± 2^**^	8.1 ± 0.8	6.5 ± 1.0^*^	74 ± 15	45 ± 2^*^
**ACTIVITIES**								
Cytochrome *c* oxidase	14.3 ± 0.1	10 ± 1^**^	168 ± 28	116 ± 19^**^	27 ± 1	16 ± 1^**^	149 ± 17	64 ± 4^**^
Citrate synthase	274 ± 52	293 ± 31	2314 ± 305	2400 ± 320	263 ± 27	282 ± 39	2258 ± 360	2324 ± 199

*All activities were expressed as nmol oxygen consumed × (min × mg protein)^−1^. Citrate synthase was expressed as nmol × (min × mg protein)^−1^*.

*Statistical analysis was carried out with the Student's t-test. The p-values are as follows:*

* *< 0.05*,

** *< 0.01*.

### Isolated neurons recapitulate the mitochondrial deficits observed in KI brain regions

Non-synaptosomal mitochondria as well as mitochondria-enriched fractions are, by necessity, removed from their cytoplasmic environment with the potential of altering their function through the purification process. To address this issue, in parallel we tested mitochondrial function in a system retaining cellular specificity and with an intact intracellular milieu. OXPHOS was directly evaluated in isolated, intact hippocampal, cerebellar, and cortical neurons obtained from 4–6 pooled WT and KI mice at PND0 (Table 2). Glucose-sustained basal respiration of intact neurons, followed by the addition of the ATPase inhibitor oligomycin, was recorded to establish the ATP production linked to oxygen uptake. Subsequent addition of FCCP, an uncoupler of ATP production and electron transfer, allowed evaluating the maximum mitochondrial oxygen uptake capacity. ROS-mediated oxygen uptake and membrane proton leak (oligomycin-resistant oxygen uptake rates), oxygen uptake linked to ATP production (oligomycin-sensitive), and spare respiratory capacity (FCCP-mediated maximal respiration rate) were expressed as a fraction of the basal oxygen consumption rate for both WT and KI neurons (Table 2). At PND0, a deficiency in the ATP-driven oxygen uptake was already evident in isolated KI hippocampal neurons, consistent with this area being the most affected.

**Table 2:** **Mitochondrial coupling and respiratory capacity in intact, isolated neurons from WT and KI mice at PND0**.

**Outcomes**	**Hippocampus**		**Cerebellum**		**Cortex**	
	**(% Basal rate)**					
**Oxygen consumption rates**	**WT**	**KI**	**WT**	**KI**	**WT**	**KI**
Oligomycin-sensitive	90 ± 1	85 ± 1^**^	89 ± 3	80 ± 6	84 ± 2	77 ± 3
Oligomycin-resistant	0.33 ± 0.33	6.9 ± 0.5^***^	−0.22 ± 0.22	0.9 ± 0.2^*^	3 ± 5	19 ± 3^*^
Spare respiratory capacity	128 ± 12	62 ± 2^**^	102 ± 30	74 ± 42	110 ± 17	67 ± 12
**Coupling**	**Hippocampus**		**Cerebellum**		**Cortex**	
RCRu	11 ± 2	3.4 ± 0.8^*^	9 ± 1	1.7 ± 0.4^**^	7 ± 1	0.9 ± 0.3^***^

*Oxygen consumption rates under basal conditions (in the presence of glucose only) were not statistically significant different between WT and KI in any of the brain regions [average of WT and KI = 6.5 ± 0.7; 3.4 ± 0.9; and 3.1 ± 0.5 nmol oxygen consumed × (min × 10^6^ cells)^−1^ for cortex, cerebellum and hippocampus, respectively]. Oxygen uptake linked to ATP production (oligomycin-sensitive mitochondrial oxygen uptake), ROS-mediated oxygen uptake and membrane proton leak (oligomycin-resistant mitochondrial oxygen uptake), and spare respiratory capacity (FCCP-mediated maximal respiration rate) were expressed as a fraction of the basal oxygen consumption rate. The RCRu represents the ratio between FCCP and oligomycin-resistant oxygen uptake rates. Data (from three separate preparations run in duplicates) are shown as mean ± SEM. Statistical comparison between WT and KI was performed with the Student's t-test. The p-values are as follows*:

* *< 0.05*;

** *< 0.005*;

*** *< 0.0005*.

Interestingly, KI neurons show a dramatically lower spare respiratory capacity than WT, capacity defined as the mitochondrial ability to meet increased energy demand with increased respiration (Table 2). This may indicate a lower capacity to adapt to stressful situations with increased ATP need. The coupling (as judged by the RCRu) and the oligomycin-resistant oxygen uptake were significantly different (3–5-fold lower and 4–20-fold higher, respectively, than controls) in KI compared to WT in all brain areas (Table 2). The lower RCRu and spare respiratory capacity of hippocampal KI cells are of significant biological relevance because energy deficits generated by an imbalance between bioenergetic reserve and demand, play a critical role in the survival of neurons under stress conditions (Nicholls, 2008) and can lead to disrupted synaptic network (Selkoe, 2002; Yadava and Nicholls, 2007).

The apparent discrepancy between the marked deficits observed at PND0 in KI isolated neurons (Table 2) relative to the ones at PND7 in KI non-synaptosomal mitochondria (Table 1) or total mitochondrial fractions (Figure 2) can be bridged taking into account the absence of supporting cells (glia) in a neuron-only culture system (Iwata-Ichikawa et al., 1999; Bélanger and Magistretti, 2009), which could be even more critical for neurons with an already compromised genetic background. Alternatively, neuronal mitochondria might be more affected than those of glia, masking the effect when both are tested in mitochondria-rich fractions from brain.

### Coupling between electron transport and ATP synthesis correlates with FMRP but not *FMR1* expression levels in human fibroblasts

**Figure 4:**
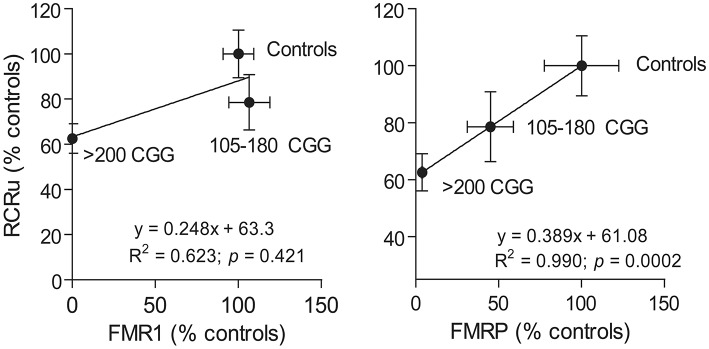
**Correlation between mitochondrial outcomes and FMRP or *FMR1* expression levels**. The correlation between FMRP and *FMR1* expression with a mitochondrial outcome (i.e., coupling between ATP synthesis and electron transfer or RCRu) was carried out using human primary dermal from controls, premutation carriers (105–180 CGG), and full mutation carriers (>200 CGG repeats). RCRu, *FMR1*, and FMRP levels were expressed as percentage of control values. Data are shown as mean ± SEM for controls, permutation, and full mutation carriers.

As a proof of concept, to test whether *FMR1* gene or FMRP protein expression influence mitochondrial outcomes, we turned to human primary skin fibroblasts from premutation and full mutation carriers, as commercially available *FMR1* KO mice are on a different genetic background (FVB129 or FVB129/NJ) than the ones used in this study (C57BL/6J) making the comparison invalid [see as an example for Huntington's disease, another triplet-nucleotide repeat disease (Menalled et al., 2009)]. In addition, KO mice may not recapitulate the full mutation seen in humans, since humans show no expression of FMRP over time as a result of a repeat-mediated gene silencing sometime during early development (Pieretti et al., 1991; Sutcliffe et al., 1992). To this end, *FMR1* and FMRP expression were assessed in fibroblasts from controls, premutation (105–180 CGG matching the range of CGG in KI mice), and full mutation (>200 CGG repeats) carriers (Figure 4). Fibroblasts from premutation carriers showed normal or marginally elevated *FMR1* transcript levels with ~50% FMRP levels of controls; in contrast, fibroblasts from full mutation carriers showed no gene or protein expression. When the link between *FMR1* and FMRP expression and bioenergetics (as judged by coupling between ATP synthesis and electron transfer, RCRu) was investigated in these samples, a strong direct correlation was observed between RCRu and FMRP levels but not *FMR1* mRNA levels (Figure 4).

Further studies are warranted by including either a wider array of outcomes or more subjects, however, these results are consistent with the association between bioenergetics and FMRP protein expression rather than to *FMR1* transcript levels.

### Zn concentrations, bioenergetics, and Shank3 deficiencies in KI brain

The bioenergetic deficits observed in brains from KI mice were similar to those reported in previous studies performed on primary dermal fibroblasts from older premutation carriers (Napoli et al., 2011). That study ascribed the bioenergetic deficits to altered ZnT6 protein level and lower transport of cytoplasmic Zn (Napoli et al., 2011), which resulted in deficient Zn-mediated import/processing of nuclearly-encoded mitochondrial subunits (Napoli et al., 2011). As a result of the defective Zn-dependent processing/import of nuclear DNA (nDNA)-encoded mitochondrial proteins, higher ratios of precursor-to-mature mitochondrial proteins were observed in fibroblasts and brain samples of premutation carriers.

**Figure 5:**
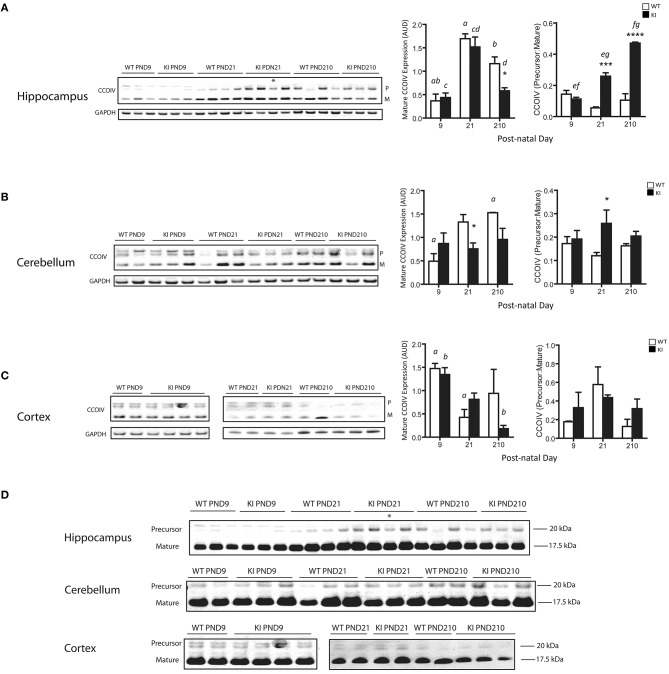
**Protein expression of precursor and mature CCOIV in brains from KI mice**. Representative Western blots of CCOIV (precursor and mature proteins) in hippocampus **(A)**, cerebellum **(B)** and cortex **(C)** of WT and KI mice. The densitometry for all the samples is also shown. Data were expressed as Arbitrary Densitometry Units and reported as mean ± SEM. Mature form of CCO4 was normalized by GAPDH. Data are reported as mean ± SEM, *n* = 3–5 per genotype per time point, ran in triplicates. Statistical analysis was performed by Two-way ANOVA, followed by Tukey *post-hoc* test for multiple comparisons. Statistically significant differences between WT and KI are indicated by asterisks as follows. Hippocampus: ^*^p = 0.0416; ^***^p = 0.0002; ^****^p < 0.0001. Cerebellum: ^*^p = 0.0212 for mature CCO at PND21; ^*^p = 0.0197 for P:M at PND21. Statistically significant differences among time points are indicated by letters as follows. Hippocampus: *p* = 0.0002 (*a*), *p* = 0.0172 (*b*), *p* = 0.0013 (*c*), *p* = 0.0047 (*d*), *p* = 0.0086 (*e*), *p* < 0.0001 (*f*), *p* = 0.0003 (*g*). Cerebellum: *p* = 0.0459 (*a*). Cortex: *p* = 0.0391 (*a*), *p* = 0.0113 (*b*). Further statistical details on the genotype, age, and genotype × age effect can be found in Table 3. **(D)** Uncropped version of the Western blot image showing the intensity and mobility of the precursor band relative to the mature protein. The observed molecular weights for the CCOIV precursor and mature forms were, respectively, 20.0 and 17.5 kDa, as extrapolated by the Molecular Weight markers with the use of the Carestream software. This 2.5 kDa difference was close to the theoretical calculated molecular weight (2.4 kDa) of the 22 residues of amino acids (MLATRVFSLVGKRAISTSVCVR) present in the precursor form, which is cleaved by mitochondrial matrix peptidases (Isaya et al., 1991) to produce the mature mitochondrial form of the protein (UniProtKB P13073). Asterisk in the immunoblot image of hippocampus (panels A and D) denotes a 90 CGG KI sample. Densitometry data for this sample have not been included in the averages for KI. For cortex, samples were run in two separate gels (PND9 and PND21-210). AUD, Arbitrary Units of Densitometry.

To test whether the import/processing of nDNA-encoded mitochondrial proteins was also affected in the KI mouse model, providing an explanation for the observed defects in OXPHOS, we evaluated the protein expression levels of mature and precursor cytochrome *c* oxidase subunit IV (CCOIV, Figure 5) in brains from WT and KI mice. The CCOIV Precursor-to-Mature ratios (P:M) were significantly increased in hippocampus (PND21 and PND210) and cerebellum (PND21) of KI mice (Figure 5), consistent with our previous reports.

**Figure 6:**
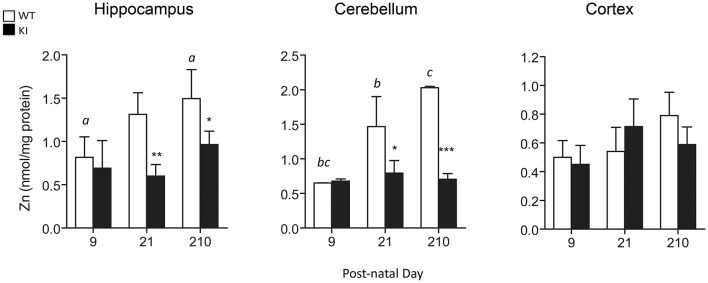
**Brain Zn concentrations in WT and KI mice**. Total Zn levels were measured in whole hippocampus, cerebellum and cortex homogenates from WT and KI mice. Data are shown as mean ± SEM, *n* = 3–6 individuals *per* genotype, *per* time point, ran individually. Statistical analysis was performed by Two-way ANOVA, followed by Tukey's HSD post-hoc test for multiple comparisons. Statistically significant differences between WT and KI at individual time-points are indicated by asterisks as follows: Hippocampus: ^*^p = 0.0425; ^**^p = 0.0070; Cerebellum: ^*^p = 0.0158; ^***^p = 0.0003. Statistically significant differences among time points are indicated by letters as follows: *p* = 0.0190 (*a*), *p* = 0.0144 (*b*), *p* = 0.0003 (*c*). Further statistical details on the genotype, age, and genotype × age effect can be found in Table 3.

**Figure 7:**
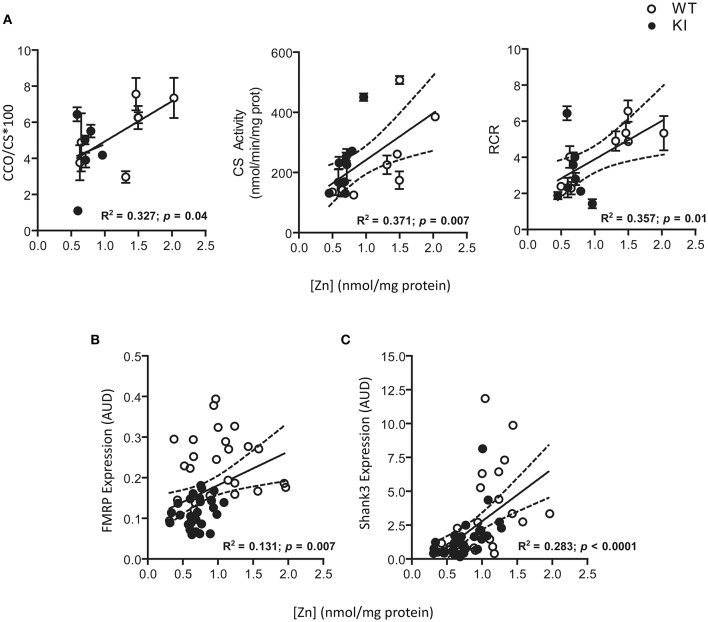
**Correlations among Zn levels, selected mitochondrial outcomes, and protein expression of FMRP and Shank3, in brain of WT and KI mice**. A significant direct correlation was noted between average Zn levels and activities of CCO and CS and RCR for all the time points analyzed **(A)**. CCO and CS activities were expressed as nmol × (min × mg protein)^−1^. CCO activity has been normalized to citrate synthase and multiplied by 100. All the other mitochondrial outcomes did not show a correlation with Zn. A direct correlation was also observed between Zn levels and both FMRP and Shank3 protein expression (individual values) in brains of WT and KI when all time points were combined **(B,C)**. Dotted lines illustrate the 95% CI obtained with WT and KI values.

To test for Zn homeostasis, Zn levels in brain were evaluated in parallel. Lower Zn concentrations were observed in hippocampus and cerebellum of KI mice at PND21 and lasting into PND210 for both brain regions (Figure 6). Interestingly, these regions are the ones with the highest Zn concentrations (average two-fold of cortex; Figure 6), consistent with their higher fraction of Zn-containing neurons (Sawashita et al., 1997) and neuron-to-glia ratios (Napoli et al., 2012). In agreement with the findings previously obtained with fibroblasts from older premutation carriers (Napoli et al., 2011), a positive correlation was also observed between Zn levels and mitochondrial outcomes (namely, activities of Complex IV and citrate synthase and coupling; Figure 7). Taken together, these results confirmed the lower import/processing capacity of nDNA-encoded proteins to mitochondria in premutation and the occurrence of altered Zn bioavailability.

Zn deficits are linked not only to altered mitochondrial protein import (Tokatlidis et al., 2000; Napoli et al., 2011) but also to dysregulation of Shank2/3 scaffolding (Grabrucker et al., 2014). Indeed, Shank3 transcript—as actin transcript—has been identified as one of the main mRNAs interacting with FMRP (Darnell et al., 2011) and both FMRP and *FMR1* mRNA are normally present in ribosomes associated to postsynaptic dendritic sites, location shared by Shank3 (Weiler et al., 1997).

**Figure 8:**
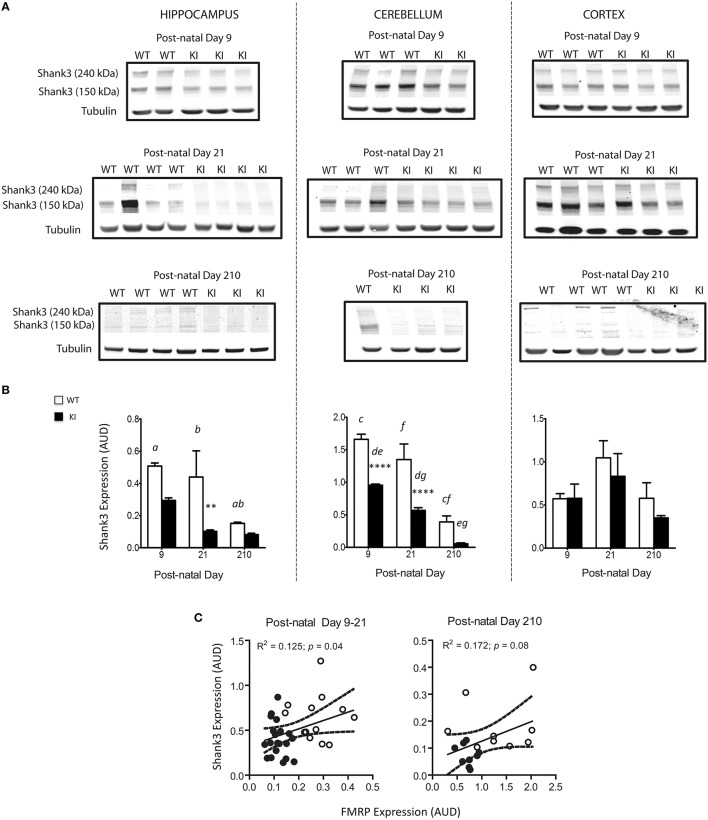
**Brain Shank3 protein levels in WT and KI mice. (A)** Western blots for Shank3 protein expression levels in hippocampus, cerebellum, and cortex were performed at PND 9, 21, and 210 from WT (white) and KI (black) mice as described in details in the Materials and Methods Section. Further, images taken with acetone-precipitated samples and normalized by either tubulin or GADPH are found under Supplementary Figure 1. **(B)** Densitometry data is shown as mean ± SEM, *n* = 4–5 individuals *per* genotype, *per* time point, ran individually. Statistical analysis was performed by two-way ANOVA, followed by Tukey's HSD *post-hoc* test for multiple comparisons. Statistically significant differences between WT and KI are indicated by asterisks as follows: Hippocampus: ^**^p = 0.0054; Cerebellum: ^****^p < 0.0001. Statistically significant differences among time points are indicated by letters as follows: *p* = 0.0074 (*a*), *p* = 0.0267 (*b*), *p* < 0.0001 (*c, e, f*), *p* = 0.0217(*d*), *p* = 0.0041 (*g*). Further, statistical details on the genotype, age, and genotype × age effect can be found in Table 3. **(C)** Correlation between FMRP levels and Shank3 protein expression in brain of WT and KI mice. Both proteins have been normalized by tubulin, used as loading control. Shown are individual data points collected in cerebellum, hippocampus, and cortex of WT and KI mice at PND9, PND21, and PND210. PND210 is shown in a separate plot due to the difference in protein expression of both FMRP and Shank3 at this time point, relative to the previous ones. Dotted lines are 95% CI constructed with WT and KI values. AUD, Arbitrary Units of Densitometry.

Deficits in Shank3 protein expression—evaluated by Western blots—were noticeable at PND9, PND21, and PND210 in hippocampus and cerebellum from KI mice (Figure 8 and Supplementary Figure 1). These results are consistent with other studies reporting Shank3 protein expression being brain-region/cell-type specific and developmentally regulated (Wang et al., 2014). Of note, although Shank3 is a postsynaptic density protein, several studies have shown that Shank3 expression pattern in whole brain areas mirrors that observed at the synaptic regions (Han et al., 2013; Kouser et al., 2013) providing support for the Shank3 expression in total homogenates from brain regions. Furthermore, the amount of tissue collected from each pup, especially at early time points—i.e., PND9 and PND21—would not have been sufficient as starting material for postsynaptic density preparations.

Given that Zn deficits were observed after those in Shank3, it could be inferred that the reduced levels of FMRP at early time points affects mainly Shank3 translation whereas later, both Shank3 protein expression and Zn-dependent scaffolding seem affected. In brains of WT and KI mice, statistically significant positive correlations were noted between FMRP and Shank3 (Figure 8C), FMRP and Zn levels (Figure 7B) and between Shank3 and Zn levels (Figure 7C), reinforcing the concept of the crosstalk between FMRP, Shank3, and Zn homeostasis.

### Interaction between genotype and age on FMRP levels, bioenergetics, Zn, and Shank3 in brains of WT and KI mice

To test the putative interaction between genotype and age, a Two-way ANOVA analysis was performed for each of the outcomes tested at different time-points (Table 3). A statistically significant genotype × age interaction was observed in hippocampus for FMRP, most of the mitochondrial outcomes tested (five of eight; NADH oxidase activity, uncoupling, citrate synthase activity, and ATPB and COXIV expression), and Shank3, whereas in cerebellum the interaction was statistically significant for FMRP, only one mitochondrial outcome (COXIV), Zn levels and Shank3 (Table 3). In cortex, the interaction of age and genotype was statistically significant only for FMRP levels (Table 3). Thus, the tissue that showed the most evident gene × age interaction was hippocampus, followed by cerebellum and then cortex (Table 3). Simple main effect analysis showed that the overall changes observed in hippocampus and cerebellum could be equally attributed to genotype and age, whereas in cortex age had a more prominent effect (Table 3).

**Table 3:** **Effect of genotype, age, and genotype × age interaction on outcomes evaluated in hippocampus, cerebellum, and cortex of WT and KI mice**.

	**Genotype × age interaction**	**Genotype effect**	**Age effect**
**HIPPOCAMPUS**			
FMRP	*F*_(2, 17)_ = 23.42	*F*_(1, 17)_ = 105.3	F_(2, 17)_ = 16.24
	***p*** < **0.0001**	***p*** < **0.0001**	***p*** = **0.0001**
NADH oxidase	*F*_(2, 18)_ = 8.466	*F*_(1, 18)_ = 2.435	*F*_(2, 18)_ = 17.12
	***p*** = **0.0026**	***p*** = **0.0464**	***p*** < **0.0001**
Succinate oxidase	*F*_(2, 19)_ = 0.6392	*F*_(1, 19)_ = 6.180	*F*_(2, 19)_ = 16.02
	*p* = 0.5387	***p*** = **0.0224**	***p*** < **0.0001**
Cytochrome *c* oxidase	*F*_(2, 18)_ = 1.099	*F*_(1, 18)_ = 5.245	*F*_(2, 18)_ = 2.915
	*p* = 0.3545	***p*** = **0.0343**	*p* = 0.0801
RCR	*F*_(2, 22)_ = 9.284	*F*_(1, 22)_ = 27.45	*F*_(2, 22)_ = 8.578
	***p*** = **0.0012**	***p*** < **0.0001**	***p*** = **0.0018**
Citrate synthase	*F*_(3, 47)_ = 1.307	*F*_(1, 47)_ = 2.162	*F*_(3, 47)_ = 8.239
	*p* = 0.283	*p* = 0.1481	***p*** = **0.0002**
ATPB	*F*_(2, 15)_ = 26.74	*F*_(1, 15)_ = 214.1	*F*_(2, 15)_ = 39.95
	***p*** < **0.0001**	***p*** < **0.0001**	***p*** < **0.0001**
COXIV	*F*_(2, 15)_ = 2.362	*F*_(1, 15)_ = 3.582	*F*_(2, 15)_ = 34.88
	*p* = 0.1283	*p* = 0.0779	***p*** < **0.0001**
COXIV (P:M)	*F*_(2, 15)_ = 30.14	*F*_(1, 15)_ = 78.45	*F*_(2, 15)_ = 23.12
	***p*** < **0.0001**	***p*** < **0.0001**	***p*** < **0.0001**
[Zn]	*F*_(2, 17)_ = 2.588	*F*_(1, 17)_ = 19.94	*F*_(2, 17)_ = 7.303
	*p* = 0.1045	***p*** = **0.0003**	***p*** = **0.0051**
Shank3	*F*_(2, 20)_ = 2.559	*F*_(1, 20)_ = 16.50	*F*_(2, 20)_ = 10.23
	***p*** = **0.1024**	***p*** = **0.0006**	***p*** = **0.0009**
**CEREBELLUM**			
FMRP	*F*_(2, 15)_ = 4.768	*F*_(1, 15)_ = 28.64	*F*_(2, 15)_ = 13.16
	***p*** = **0.0250**	***p*** < **0.0001**	***p*** = **0.0005**
NADH oxidase	*F*_(3, 48)_ = 1.471	*F*_(1, 48)_ = 0.08211	*F*_(3, 48)_ = 6.907
	*p* = 0.2341	*p* = 0.7757	*p* = **0.0006**
Succinate oxidase	*F*_(3, 44)_ = 0.2040	*F*_(1, 44)_ = 1.196	*F*_(3, 44)_ = 8.347
	*p* = 0.893	*p* = 0.2802	***p*** = **0.002**
Cytochrome *c* oxidase	*F*_(3, 42)_ = 1.198	*F*_(1, 42)_ = 0.1964	*F*_(3, 42)_ = 2.490
	*p* = 0.3221	*p* = 0.6559	*p* = 0.0733
RCR	*F*_(3, 40)_ = 1.493	*F*_(1, 40)_ = 4.457	*F*_(3, 40)_ = 1.044
	*p* = 0.2312	***p*** = **0.0411**	*p* = 0.3835
Citrate synthase	*F*_(3, 57)_ = 7.890	*F*_(1, 57)_ = 0.9931	*F*_(3, 57)_ = 43.11
	*p* = 0002	*p* = 0.2323	***p*** < **0.0001**
ATPB	*F*_(2, 12)_ = 1.194	*F*_(1, 12)_ = 7.348	*F*_(2, 12)_ = 5.603
	*p* = 0.3427	***p*** = **0.0219**	***p*** = **0.0233**
COXIV	*F*_(2, 12)_ = 5.355	*F*_(1, 12)_ = 4.313	*F*_(2, 12)_ = 4.394
	***p*** = **0.0262**	*p* = 0.0645	***p*** = **0.0427**
COXIV (P:M)	*F*_(2, 12)_ = 3.523	*F*_(1, 12)_ = 8.816	*F*_(2, 12)_ = 0.3843
	*p* = 0.0695	***p*** = **0.0141**	*p* = 0.6906
[Zn]	*F*_(2, 11)_ = 12.09	*F*_(1, 11)_ = 37.88	*F*_(2, 11)_ = 13.61
	***p*** = **0.0017**	***p*** < **0.0001**	***p*** = **0.0011**
Shank3	*F*_(2, 18)_ = 4.293	*F*_(1, 18)_ = 85.27	*F*_(2, 18)_ = 95.15
	***p*** = **0.0299**	***p*** < **0.0001**	***p*** < **0.0001**
**Cortex**			
FMRP	*F*_(2, 12)_ = 9.336	*F*_(1, 12)_ = 38.50	*F*_(2, 12)_ = 14.01
	***p*** = **0.0036**	***p*** < **0.0001**	***p*** = **0.0007**
NADH oxidase	*F*_(3, 41)_ = 0.2289	*F*_(1, 41)_ = 0.0008	*F*_(3, 41)_ = 19.52
	*p* = 0.8757	P = 0.9286	***p*** < **0.0001**
Succinate oxidase	*F*_(3, 46)_ = 0.09088	*F*_(1, 46)_ = 1.045	*F*_(3, 46)_ = 15.24
	*p* = 0.9647	*P* = 0.3120	***p*** < **0.0001**
Cytochrome *c* oxidase	*F*_(3, 46)_ = 0.09514	*F*_(1, 46)_ = 3.203	*F*_(2, 46)_ = 12.30
	*p* = 0.9623	*p* = 0.0801	***p*** < **0.0001**
RCR	*F*_(3, 45)_ = 0.2112	*F*_(1, 45)_ = 4.890	*F*_(3, 45)_ = 2.388
	*p* = 0.8881	***p*** = **0.0321**	*p* = 0.0813
Citrate synthase	*F*_(3, 55)_ = 1.983	*F*_(1, 55)_ = 1.983	*F*_(3, 55)_ = 213.7
	*p* = 0.1272	*p* = 0.3405	***p*** < **0.0001**
ATPB	*F*_(2, 11)_ = 1.249	*F*_(1, 11)_ = 0.6066	*F*_(2, 11)_ = 15.49
	*p* = 0.3246	*p* = 0.4525	***p*** = **0.0006**
COXIV	*F*_(2, 12)_ = 3.819	*F*_(1, 12)_ = 1.054	*F*_(2, 12)_ = 12.72
	*p* = 0.0630	*p* = 0.3313	***p*** = **0.0024**
COXIV (P:M)	*F*_(2, 12)_ = 1.018	*F*_(1, 12)_ = 0.4778	*F*_(2, 12)_ = 3.047
	*p* = 0.3993	*p* = 0.5069	*p* = 0.0976
[Zn]	*F*_(2, 12)_ = 1.913	*F*_(1, 12)_ = 0.1174	*F*_(2, 12)_ = 3.011
	*p* = 0.1979	*p* = 0.7389	*p* = 0.0947
Shank3	*F*_(2, 12)_ = 0.4705	*F*_(1, 12)_ = 1.679	*F*_(2, 12)_ = 9.085
	*p* = 0.6322	*p* = 0.2114	***p*** = **0.0019**

*Statistical parameters reported in the table were calculated by Two-way ANOVA. In bold are statistically significant effects. Between parentheses are values of F(DFn, DFd). Further, statistical details are reported in the figure legend of each outcome tested*.

In terms of outcomes, FMRP expression was the only parameter that showed a strong interaction between genotype and age in all three-brain regions, with a bigger contribution of genotype than age (Figure 1 and Table 3). Differences recorded in mitochondrial outcomes appeared to be determined by both genotype and age in hippocampus, while age played a bigger role in cerebellum and cortex (Figure 2 and Table 3). Differences observed in Zn levels in hippocampus and cerebellum seemed mainly due to genotype more than age (Figure 6 and Table 3). Finally, Shank3 expression seemed to be influenced by genotype and age in hippocampus and cerebellum (Figure 8 and Table 3), and mainly by age in cortex. Taken together, this analysis indicates a stronger effect of the KI genotype than age on the outcomes evaluated in hippocampus, about equal for cerebellum and mostly age-dependent effects in cortex.

### Altered ZnT gene expression in lactating mammary glands from KI dams

While some of the Zn, Shank3, and mitochondrial deficits lasted into PND210 in hippocampus and cerebellum (Figures 2–8), the finding that most of the mitochondrial outcomes were significantly different at PND21 (at the end of the nursing period) and that some amelioration was observed between PND21 and PND210 in cortex and cerebellum, suggested that lactation (nursing from KI dams vs. post-weaning diet constituted by vitamin- and mineral-balanced murine chow) might have compounded the early deficits. Due to the unsuccessful attempts to collect sufficient murine milk to evaluate Zn levels or any other biochemical analyses, as a surrogate for Zn homeostasis we tested the gene expression of *ZnT4* and *ZnT6* in lactating mammary glands from control and KI dams. *ZnT4* and *ZnT6* gene expression was evaluated by PCR, followed by separation of the products in a 1.3% agarose gel, visualized by using ethidium bromide. (Commercially available dual-labeled probes for these transporters resulted unspecific).

**Figure 9:**
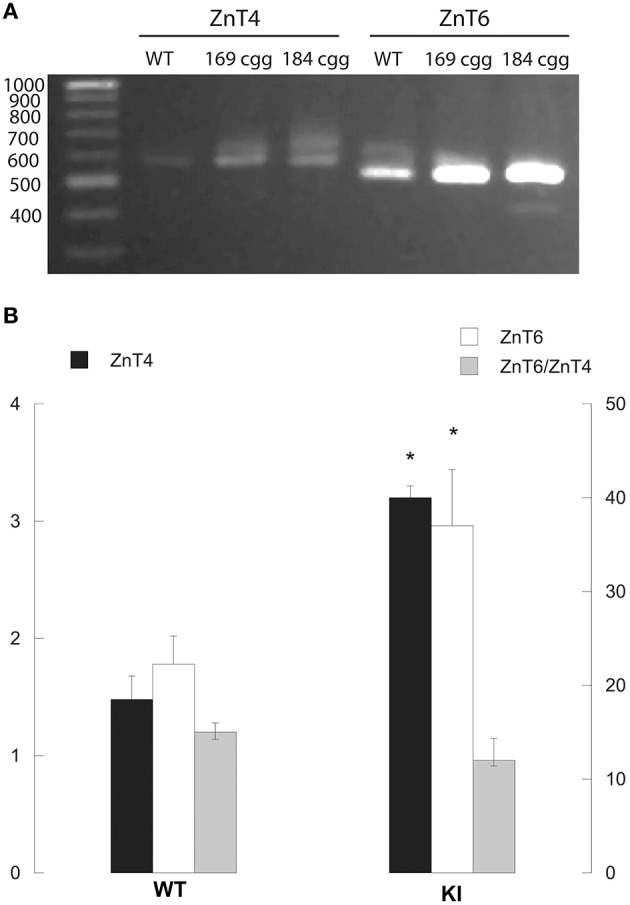
***ZnT4* and *ZnT6* gene expression in mammary glands from lactating WT and KI dams**. Representative image of the PCR products (obtained by RT-qPCR as described under Methods) that were separated in a 1.3% agarose gel and visualized with ethidium bromide **(A)**. The fragments exhibited the expected sizes of 585 bp (*ZnT4*) and 537 bp (*ZnT6*). *GAPDH* PCR product was separated in a 3% agarose gel electrophoresis with ethidium bromide resulting in a product of the expected of 107 bp (not shown). Intensities of *ZnT4* and *ZnT6* bands were obtained in a Kodak Imager were normalized by that of *GAPDH* **(B)**. Bars represent averages ± SEM of 4 individuals/genotype. Statistical analysis was carried out with the Student's *t*-test between WT and KI. ^*^p < 0.05.

In lactating mammary glands from WT dams, both *ZnT4* and *ZnT6* (normalized to *GAPDH*) were expressed with a ratio of *ZnT6/ZnT4* equal to 15 (Figure 9A). The gene expression of both ZnTs was significantly higher (two-fold) in KI than WT, with no significant changes in the *ZnT6/ZnT4* expression ratio compared to WT (Figure 9B). These results suggested a disrupted ZnT expression, which may affect Zn efflux in milk, and as a consequence, the Zn status of the suckling pups.

Studies have shown that Zn deficiency in rodents results in anorexia (with about 50% of the body weight gained under physiological conditions), poor hair coat, scaly paws, and reproductive defects (Brody, 1999), none of which have been observed in either KI dams or pups, supporting the idea of a localized Zn homeostasis deficit related to the premutation (probably linked to the tissue expression of FMRP), rather than a generalized one.

### Detrimental effect of KI milk on brain bioenergetics

**Figure 10:**
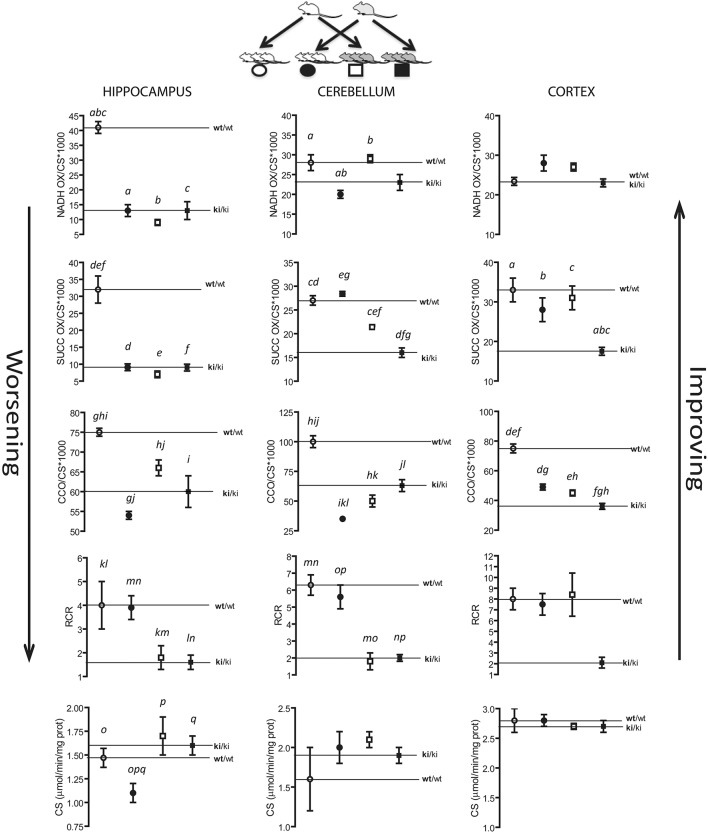
**Changes in mitochondrial outcomes in hippocampus, cerebellum and cortex of suckling WT and KI pups nursed on WT or KI dams**. At birth, KI pups (*n* = 24) and WT pups (*n* = 24) were foster-nursed either on KI dams or WT dams, six pups on each dam. After 21 days, mitochondria were isolated from cortex, cerebellum, and hippocampus and activities of NADH oxidase, succinate oxidase, cytochrome *c* oxidase, and citrate synthase and RCR were evaluated as described in the Methods section. wt/wt = WT pups nursing on WT milk; ki/ki = KI pups nursing on KI milk. Circles represent WT pups, squares represent KI pups. White symbols represent WT milk and black symbols represent KI milk. Upward or downward arrows represent improvement or worsening, respectively, upon nursing on WT milk or KI milk. Activities (NADH oxidase, succinate oxidase, cytochrome *c* oxidase) were expressed as nmol × (min × mg protein)^−1^, normalized by citrate synthase activity and multiplied by 1000. Data are shown as mean ± SEM (from technical replicates of pooled samples). Statistical analysis was performed by Two-way ANOVA, followed by Tukey's *post-hoc* test for multiple comparisons. The *p* values are as follows. Hippocampus: *p* < 0.0001 (*a, b, c, g, i, p, q*), *p* = 0.0004 (*d, o*), *p* = 0.0002 (*e, f*), *p* = 0.0025 (*h*), *p* = 0.0008 (*j*), *p* = 0.0027 (*k*), *p* = 0.0006 (*l*), *p* = 0.0262 (*m*), *p* = 0.0082 (*n*); Cerebellum: *p* = 0.0296 (*a*), *p* = 0.0301 (*b*), *p* = 0.0039 (*c*), *p* < 0.0001 (*d, g, h, i, j, k, l, m, n, o, p*), *p* = 0.0023 (*e*); *p* = 0.0086 (*f*); Cortex: *p* < 0.0001 (*a, b, c, d, e, f*), *p* = 0.0047 (*g*), *p* = 0.0468 (*h*). Further statistical details on the genotype, age, and genotype × age effect can be found in Table 4.

On the basis of the findings shown thus far we hypothesize that Zn-deficient milk of KI dams would be detrimental on the suckling pups' bioenergetics requirements, whereas milk from WT dams might rescue some of the deficits observed in the premutation mice. To test this hypothesis we designed a cross-fostering experiment in which KI pups nursed on WT dams (WT milk) and WT pups nursed on KI dams (KI milk; Figure 10). We tested mitochondrial outcomes at PND21 from WT and KI in the pups (male hemizygous) nursing on KI milk to elucidate the effect of KI milk on the brain bioenergetics of WT pups, or nursing on WT milk to elucidate the effect of WT milk on the brain bioenergetics of KI pups (Figure 10).

A detrimental effect on brain bioenergetics was observed in WT pups nursing on KI milk, characterized by significantly lower mitochondrial outcomes relative to WT pups nursing on WT milk in all three brain areas, with the highest effect observed in hippocampus showing, deficits in four of the five measured outcomes. On average, the most affected outcome was ATP-dependent oxygen uptake sustained by NAD-linked substrate (NADH oxidase activity), followed by ATP-dependent oxygen uptake sustained by an FAD-linked substrate (succinate oxidase activity), CCO activity, and coupling (Figure 10). Furthermore, the hippocampus of WT pups nursing on KI milk showed a significant decrease in mitochondrial mass. Of note, although the detrimental effects of the KI milk on the mitochondrial outcomes in WT pups were significant (–29 ± 8% average of all outcomes), they were not severe enough to match the deficits observed in KI pups nursed on KI milk (–46 ± 11%, mean ± SEM). KI pups nursing on WT milk compared to KI pups nursed on KI milk exhibited some mitochondrial improvement, as judged by a partial recovery of succinate oxidase activity in cerebellum (from 60 to 82% of WT) and complete recovery of succinate oxidase and coupling in cortex (Figure 10). None of the measured outcomes showed a significant recovery in hippocampus (Figure 10).

The effect of the diet (KI vs. WT milk) and genotype on mitochondrial outcomes was evaluated by Two-way ANOVA (Table 4). A significant milk type × genotype interaction on bioenergetics was observed in all brain regions, with the main effects being attributed to genotype in hippocampus and cerebellum, and to both genotype and milk type in cortex (Table 4).

**Table 4:** **Effect of genotype, diet, and genotype × diet interaction on outcomes evaluated in hippocampus, cerebellum, and cortex of cross-fostered WT and KI pups**.

	**Genotype × diet interaction**	**Genotype effect**	**Diet effect**
**HIPPOCAMPUS**			
NADH oxidase	*F*_(1, 11)_ = 58.03	*F*_(1, 11)_ = 57.52	*F*_(1, 11)_ = 32.05
	***p*** < **0.0001**	***p*** < **0.0001**	***p*** = **0.0001**
Succinate oxidase	*F*_(1, 11)_ = 20.96	*F*_(1, 11)_ = 20.49	*F*_(1, 11)_ = 14.15
	***p*** = **0.0008**	***p*** = **0.0009**	***p*** = **0.0031**
Cytochrome *c* oxidase	*F*_(1, 11)_ = 29.96	*F*_(1, 11)_ = 1.501	*F*_(1, 11)_ = 97.53
	***p*** = **0.0002**	*p* = 0.2462	***p*** < **0.0001**
RCR	*F*_(1, 11)_ = 0.1242	*F*_(1, 11)_ = 39.29	*F*_(1, 11)_ = 1.039
	*p* = 0.7312	***p*** < **0.0001**	*p* = 0.3298
Citrate synthase	*F*_(1, 11)_ = 10.35	*F*_(1, 11)_ = 26.72	*F*_(1, 11)_ = 67.89
	***p*** = **0.0082**	***p*** = **0.0003**	***p*** < **0.0001**
**CEREBELLUM**			
NADH oxidase	*F*_(1, 11)_ = 0.6863	*F*_(1, 11)_ = 15.68	*F*_(1, 11)_ = 1.384
	*p* = 0.4250	***p*** = **0.0022**	*p* = 0.2642
Succinate oxidase	*F*_(1, 11)_ = 12.75	*F*_(1, 11)_ = 97.54	*F*_(1, 11)_ = 5.300
	***p*** = **0.0044**	***p*** < **0.0001**	***p*** = **0.0419**
Cytochrome *c* oxidase	*F*_(1, 11)_ = 905.1	*F*_(1, 11)_ = 69.16	*F*_(1, 11)_ = 403.2
	***p*** < **0.0001**	***p*** < **0.0001**	***p*** < **0.0001**
RCR	*F*_(1, 11)_ = 14.28	*F*_(1, 11)_ = 1146	*F*_(1, 11)_ = 4.768
	***p*** = **0.0031**	***p*** < **0.0001**	*p* = 0.0515
Citrate synthase	*F*_(1, 11)_ = 1.582	*F*_(1, 11)_ = 0.8701	*F*_(1, 11)_ = 0.3943
	*p* = 0.2290	*p* = 0.3667	*p* = 0.5401
**CORTEX**			
NADH oxidase	*F*_(1, 11)_ = 98.63	*F*_(1, 11)_ = 4.235	*F*_(1, 11)_ = 0.5049
	***p*** < **0.0001**	*P* = 0.0641	*p* = 0.4921
Succinate oxidase	*F*_(1, 11)_ = 34.47	*F*_(1, 11)_ = 61.96	*F*_(1, 11)_ = 149.0
	***p*** = **0.0001**	**P**<**0.0001**	***p*** < **0.0001**
Cytochrome *c* oxidase	*F*_(1, 11)_ = 16.45	*F*_(1, 11)_ = 109.7	*F*_(1, 11)_ = 71.65
	***p*** = **0.0019**	***p*** < **0.0001**	***p*** < **0.0001**
RCR	*F*_(1, 11)_ = 60.13	*F*_(1, 11)_ = 44.72	*F*_(1, 11)_ = 84.89
	***p*** < **0.0001**	***p*** < **0.0001**	***p*** < **0.0001**
Citrate synthase	*F*_(1, 11)_ = 2.068	*F*_(1, 11)_ = 0.1195	*F*_(1, 11)_ = 0.0441
	*p* = 0.1724	*p* = 0.7347	*p* = 0.8367

*Statistical parameters reported in the table were calculated by Two-way ANOVA. Outcomes were measured in 24 mice (12 WT and 12 KI). Due to the scarce amount of tissue collected from hippocampus and cerebellum, material from four animals belonging to the same genotype and nursed from the same dam were pooled. The cross-fostering experiment was repeated twice, for a total of 48 mice used. In bold are statistically significant effects. Between parentheses are values of F(DFn, DFd). Further, statistical details are reported in the figure legend of each outcome tested*.

Taken together these results suggest that, despite the presence of a susceptible genetic background conferred by the *FMR1* premutation, brain mitochondrial outcomes were modulated by a nutritional intervention (KI milk) mainly in cortex, the least Zn-enriched brain region. While the impact of cross-fostering on offspring's brain bioenergetics could not be explained by differences in caloric intake because there were no statistical differences in body weight gain or brain weight gain between the two groups (not shown), the changes in mitochondrial brain OXPHOS in both WT and KI mice were consistent with a compounding effect of an altered Zn homeostasis in milk from premutation carriers (Table 4). While WT pups nursing on KI milk resulted in MD (hippocampus and cerebellum), KI pups nursing on WT milk improved some of the mitochondrial outcomes (cortex). The lack of a significant improvement in all of the brain regions tested could be explained by their different brain Zn requirements (Sawashita et al., 1997), limitations of a non-optimized intervention on a specific genetic background, or inability of achieving a complete recovery of some of the components involved in post-synaptic scaffolding and bioenergetics beyond PND21.

### Zn deficits in breast milk from premutation carriers

To complement and further confirm the experiments performed with the premutation mouse model, experiments were extended to test the quality of milk from lactating women carrying the premutation. Zn concentrations were determined in mature human milk [i.e., 8–20 weeks of lactation (Worth et al., 1981)] from asymptomatic premutation women (milk CGG repeats = 63–119) and age-matched controls (Table 5). Furthermore, we tested selected Zn-associated outcomes, namely activity of milk alkaline phosphatase (ALP), a Zn-requiring enzyme that contributes with 20% of total milk Zn (Fransson and Lonnerdal, 1984), and concentrations of lactose, whose biosynthesis is dependent on the Zn-requiring enzyme β-1,4-galactosyltransferase (McCormick and Kelleher, 2012), the rate limiting enzyme in the lactose biosynthetic pathway (Jagoda and Rillema, 1991).

**Table 5:** **Details of the study population**.

**Premutation carrier**	**Milk CGG repeats**	**Maternal age at sample collection (y)**	**Lactation week at sample collection**
1	29, 63	38	20
2	20, 80	34	9
3	29, 91	34	16
4	30, 93	23	19
5	29, 119	34	8
	**MEAN** ± **SEM**		
Premutation women	89 ± 9	33 ± 2	14 ± 2
(*n* = 5)			
Control women	27 ± 2	31 ± 1	13.1 ± 0.5
(*n* = 25)	(*p* = 0.002)		

*CGG repeats were evaluated by extracting total genomic DNA from milk samples and following the procedure described before (Tassone et al., 2012). The mean CGG for premutation patients represent that of the longer allele only*.

**Figure 11:**
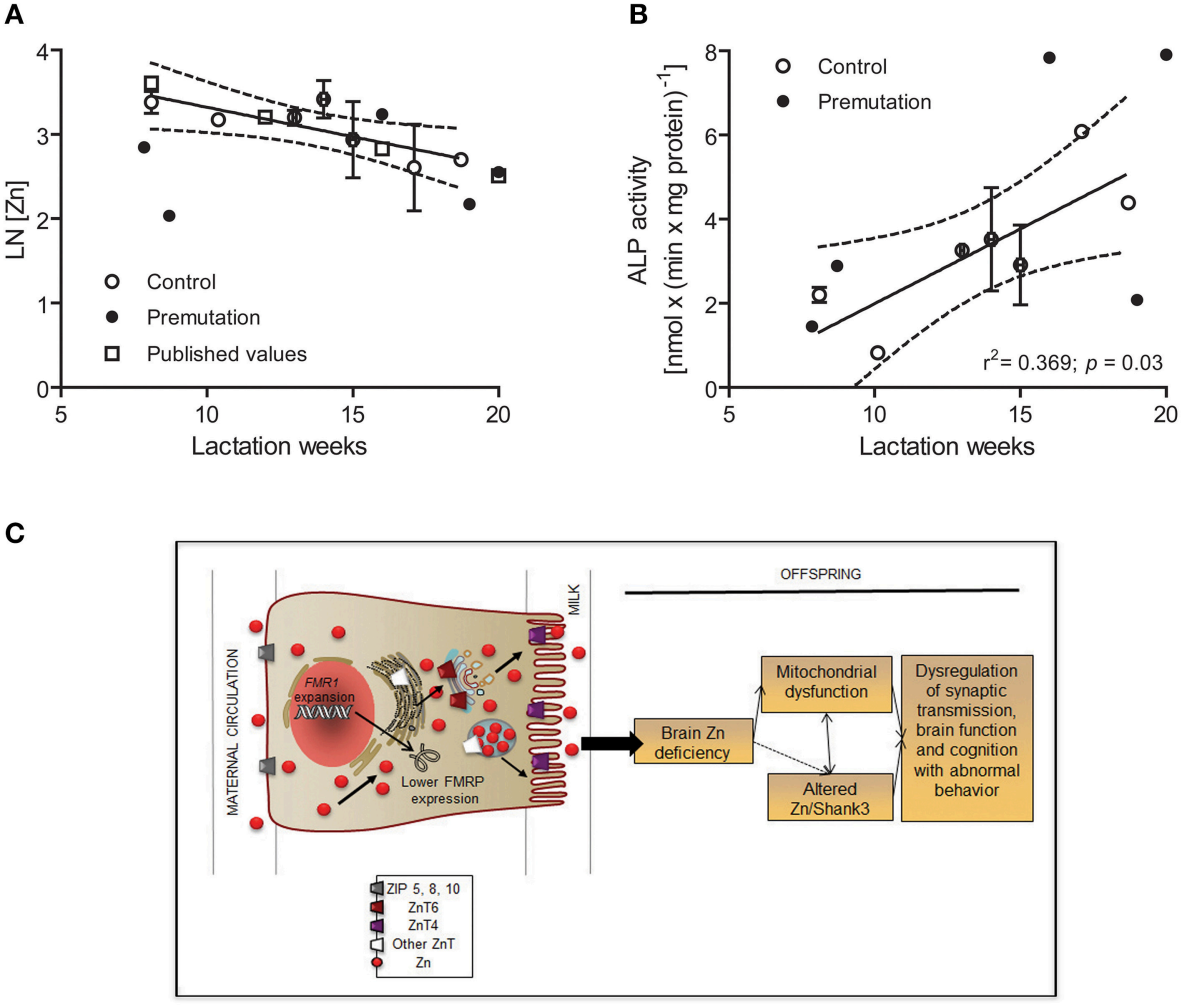
**Role of milk Zn in brain bioenergetics and synapse scaffolding in premutation carriers**. Milk-Zn concentrations followed a pseudo-first order kinetics over the lactation period **(A)**. Milk Zn concentrations from control and premutation donors decreased with the post-partum period, ranging from 30 ± 3 μM at 6–8 weeks to 15 ± 2 μM at 18–20 weeks. The LN values of the Zn concentrations (in μM) were plotted against lactation week. The published values were obtained from Nagra et al. (Nagra, 1989) and shown as white squares. Regression parameters are as follows: for published [Zn], *r*^2^ = 0.997, *y* = −0.09x + 4.3; for this study, *r*^2^ = 0.647, *y* = −0.07x + 4.0. *P* value for slopes = 0.458; *P* value for intercepts = 0.986. ALP activity [expressed as nmol × (min × mg protein)^−1^] in human breast milk from control donors increased linearly from 8 to 18–20 weeks **(B)**. The average ALP activity of controls was 3.3 ± 0.3 nmol × (min × mg protein)^−1^ or 46 ± 1 μmol × (min × l)^−1^ and within reported values [40 to 60 μmol × (min × l)^−1^; (Worth et al., 1981; Coburn et al., 1992)]. Linear regression analyses were performed with control values (reported as mean ± SEM) for both outcomes (*r*^2^ = 0.638 and 0.639 for Zn concentration and ALP activity, respectively). The 95% CIs performed with control values are shown with dotted lines. Individual premutation carriers' values for Zn concentrations and ALP activities are shown in Table 5. ALP activities in controls were not affected by vitamin and mineral supplementation [supplemented: 3.7 ± 0.4 vs. not supplemented: 3.2 ± 0.4 nmol × (min × mg protein)^−1^; *p* = 0.624]. A model integrating experimental data based on altered Zn homeostasis, mitochondrial dysfunction [current study and (Ross-Inta et al., 2010; Napoli et al., 2011)], and the putative effect of defective synaptic scaffolding in premutation individuals is shown in **(C)**. During lactation, Zn uptake is exerted by ZIP5, 8 and 10, then excreted to milk via ZnT4/ZnT2 with a lower flux through ZnT6 (ZnT10) in the Golgi [Adapted from (Kelleher et al., 2012)]. The presence of the CGG expansion in *FMR1*, as it is the case with premutation carriers, may ensue in a lower expression of FMRP, which may affect the cytoskeleton (e.g., actin, Shank3), and the expression or function of membrane transporter such as ZnT4/T6. This situation would ensue in the suboptimal delivery of Zn into milk, mitochondrial dysfunction, and synaptic dysregulation. The arrows indicate the direction of Zn transport from maternal blood to milk.

Milk from healthy donors had an average Zn concentration of 26 ± 2 μM (1.7 ± 0.1 mg/l), within the normal range reported before [1.0–1.7 mg/l at 3–5 months of lactation evaluated by atomic absorption spectrometry (Krebs et al., 1995)], with 86% of the Zn being in the labile form. In agreement with data previously reported (Nagra, 1989; Krebs et al., 1995), milk Zn concentrations from control and premutation women were reciprocally correlated with the postpartum period (Figure 11A), following a pseudo-first order kinetics with a biological half-life of 8 weeks (Figure 11). Although 10 of the 25 control donors were taking vitamins and mineral supplements (including Zn) during lactation, no significant differences in Zn concentrations were observed between these two groups, in agreement with the observed lack of correlation between milk or plasma Zn concentrations and maternal Zn intake (Krebs et al., 1995). In samples from premutation carriers, the average milk Zn concentration at 3–5 months of lactation was 56% of control values (14 ± 3 μM; *p* = 0.020). Specifically, milk Zn concentrations from two carriers at 8–9 lactation weeks and from one carrier at 19–20 weeks were significantly lower than the 95% CI (Figure 11A, Table 6). Zn levels in the remaining premutation milk samples, obtained after week 15, followed the decrease observed in control milk (Figure 11A, Table 6). Although the sample size is small, the incidence of low Zn concentration was higher in carriers compared to controls (60% vs. 24%; test for one proportion *p* = 0.0595; 95% CI = 0.2307–0.8824).

**Table 6:** **Characteristics of mature breast milk from premutation individuals**.

**Premutation carrier**	**[Zn] (μM) [mg/l]**	**ALP activity [nmol × (min × mg)^−1^]**	**[Protein] (mg/ml)**	**[Lactose] (mM)**
1	13 ± 1	7.9 ± 0.6	12.2 ± 0.4	213 ± 3
	[0.84 ± 0.08]			
2	**8** ± **2**	2.9 ± 0.3	**6.0** ± **0.1**	**160** ± **4**
	**[0.50** ± **0.05]**			
3	25 ± 2	7.3 ± 1.0	**11.4** ± **0.4**	231 ± 1
	[1.67 ± 0.13]			
4	**9** ± **1**	**2.1** ± **0.3**	17.7 ± 0.7	**196** ± **2**
	**[0.57** ± **0.09]**			
5	**17.2** ± **4**	**1.5** ± **0.1**	19.9 ± 2.0	208 ± 1
	**[1.13** ± **0.91]**			
(Mean ± SEM)	14 ± 3	4 ± 2	13 ± 3	202 ± 13
	[0.94 ± 0.21]			
<95% CI	3 of 5	1 of 5	2 of 5	2 of 5
*p*-value	0.001	n.s.	0.054	0.054
Controls (mean ± SEM)	26 ± 2	4 ± 1	12.8 ± 0.5	205 ± 3
	[1.68 ± 0.12]			
95% CI			12–13	199–210

For samples from premutation carriers, each outcome was expressed as mean ± SD, reflecting technical variability of triplicates. In bold, values below the 95% CI calculated with control values. For Zn concentrations and ALP activities, outcomes that change with the lactation week, the 95% CIs are shown in Figures 9A,B. No significant correlation was obtained between lactation week and lactose or protein concentrations, thus the 95%CIs were calculated using all control data regardless of lactation week. The p-values correspond to the test for one proportion. n.s., not significant. A post-hoc analysis to calculate the power of the analysis for the means of milk-Zn concentration from each group was 1.00, whereas for all others was 0.504 or lower (G^*^power software; v. 3.0.10).

The ALP activity in human breast milk from control donors increased almost linearly from eight to 18–20 weeks, in agreement with other reports (Chanda et al., 1951; Stewart et al., 1958; Worth et al., 1981) and with published values (Worth et al., 1981; Coburn et al., 1992; Figure 11B, Table 6). As observed with milk Zn concentrations, ALP activities in controls were not affected by vitamin supplementation. Changes in milk ALP activity with lactation week from premutation donors paralleled those of controls, with only one carrier showing a 51% ALP activity of control values at 19 weeks (Figure 11B, Table 6).

In terms of protein and lactose concentrations, the mean nutrient composition of milk samples was not different between controls and premutation individuals and remained fairly constant throughout the evaluated period, consistent with published concentrations for controls [[protein] in g/l: this study = 12.8 ± 0.5, 95%CI 12-13, literature = 12 ± 1.5 (Tudehope, 2013); [lactose] in mM: this study = 205 ± 3 95%CI 199-210, literature = 196 ± 15 (Nagra, 1989; Mohammad et al., 2012; Tudehope, 2013)]. However, the incidence of either low protein or low lactose concentrations was higher in premutation carriers than controls (Table 6). Interestingly, these observations are consistent with the findings obtained in HC11 cells in which overexpression of ZnT4 lowers both β-1,4-galactosyltransferase activity (McCormick and Kelleher, 2012) and lactose concentration by compromising the incorporation of Zn into Zn-requiring proteins.

Taken together, these results show lower milk Zn concentrations in premutation women, consistent with an altered Zn homeostasis as observed in mammary glands from KI dams, supporting the idea that adequate gene expression of *ZnT4* and *ZnT6* may be required during the switch from the non-lactating to the lactating condition to support normal Zn efflux into milk.

## Discussion

Our study reports the contribution of FMRP protein expression to the development of brain bioenergetics, cytoskeleton structure, and post-synaptic scaffolding protein Shank3 in *FMR1* premutation carriers at early stages of life. In support of this concept, we found (i) mitochondrial deficits at PND0 in isolated neurons from hippocampus, cerebellum and cortex of KI pups likely reflecting an embryonic (pre-natal) FMRP-dependent dysfunction; (ii) deficits in bioenergetics, Zn concentrations and Shank3 protein, mainly in hippocampus and cerebellum (Zn-rich brain areas) of KI pups at PND21, with some of these deficits lasting into adulthood; and (iii) defective import/processing of nDNA-encoded mitochondrial subunits secondary to impaired Zn homeostasis. A strong genotype × age interaction was observed for most of the outcomes tested in hippocampus and cerebellum, whereas in cortex, age played a major factor.

The effect of maternal milk Zn on offspring bioenergetics highlights the influence of genetics × nutrition on the premutation supported by the altered gene expression of *ZnT6/T4* in lactating KI mammary glands, the KI milk-dependent detrimental effect on KI and WT brain bioenergetics, and the lower milk Zn content in breast milk from lactating premutation women. A highly significant milk type × genotype interaction was observed for all three-brain regions being cortex the most influenced.

Brain Zn concentrations change with age, reflecting its function as a neuromodulator and as a required element for brain development (Sawashita et al., 1997), especially in regards to synaptic transmission and bioenergetics (Ho et al., 2003; Kogan et al., 2008; Grabrucker et al., 2014; Hara et al., 2014; Picard and McEwen, 2014). Our study is consistent with the reported detrimental effect of milk Zn deficiency on perinatal outcomes in the lethal milk (*lm*) mice (Huang and Gitschier, 1997), and in agreement with the proposed role of FMRP as modulator of synaptogenesis through actions on cytoskeletal proteins, by interacting with specific mRNAs [such as Shank3 or actin (Brown et al., 2001; Darnell and Richter, 2012; Han et al., 2013)] or via the Rac1 pathway (Bardoni and Mandel, 2002). The lower milk Zn concentration found in premutation women and the altered *ZnT4/ZnT6* gene expression in lactating KI murine mammary gland may account for the effect of KI milk on the brain bioenergetics of both WT and KI offspring, but more evident in KI pups. Given the heavy reliance of brain on mitochondrial ATP as well as on *de novo* synthesis of Krebs cycle-associated neurotransmitter amino acids [i.e., Glu, Asp, and GABA (Butterworth and Heroux, 1989; Navarro et al., 2008)], mitochondrial and synaptic scaffolding deficits are likely to increase the risk for some of the neurological symptoms observed in pediatric carriers. Indeed, altered brain bioenergetics and those of the post-synaptic scaffolding protein Shank3 during perinatal periods may explain the abnormal behavior observed in KI mice later in life [12 and 24 weeks; (Van Dam et al., 2005; Hunsaker et al., 2009, 2012)], similar to the spatial processing defects observed in humans with FXTAS (Hunsaker et al., 2012).

While the relative contribution of RNA sequestration of essential factors, low FMRP expression and RAN translation (of the toxic FMRPolyG) to the premutation pathology is still unknown, this study supports the notion that that lower levels of wild-type FMRP [as observed in this study and most premutation carriers (Kenneson et al., 2001), with or without the generation of aberrant FMRP isoforms (Todd et al., 2013)] may provide a disrupted cellular background that affects cytoskeleton/scaffolding (actin, Shank3) and Zn homeostasis allowing environmental factors (e.g., quality of the breast milk) to further affect synaptogenesis and mitochondrial function early in life (Figure 11C).

In support of this concept, FMRP protein expression—and not *FMR1* mRNA levels—correlates positively with mitochondrial outcomes (ATP-driven oxygen uptake and coupling) in both the KI mouse model and primary dermal fibroblasts from premutation and full mutation carriers. Furthermore, the incidence of neurodevelopmental disorders like ASD, ADHD, anxiety, and other types of psychopathologies (Farzin et al., 2006; Tassone et al., 2012; Winarni et al., 2012; Wong et al., 2012; Battistella et al., 2013; Chonchaiya et al., 2013) observed in young carriers seem to follow the FMRP protein expression [e.g., incidence of ASD in FXS is 60% (Garcia-Nonell et al., 2008; Harris et al., 2008; D'Hulst et al., 2009; Zingerevich et al., 2009; Hagerman et al., 2010) and in premutation carriers is ~15% (Farzin et al., 2006; Chonchaiya et al., 2013)]. In this regard, and consistent with our findings, the expression levels of a subset of miRNAs involved in learning, memory and autistic behavior have been shown to be deregulated in FXTAS (Zongaro et al., 2013; Nguyen et al., 2016).

While additional studies will be necessary to estimate Zn requirements for breast-fed only premutation carriers' babies and assess potential benefits of Zn-fortified milk (while controlling for confounding factors and monitoring for potential adverse effects), our findings emphasize that early nutritional interventions to prevent MD seem critical for the management of premutation carriers at high risk of developing emotional and neurological/cognitive problems (including autism) and/or FXTAS later in life (Tassone et al., 2012; Winarni et al., 2012; Wong et al., 2012; Battistella et al., 2013; Kim et al., 2013). This is largely relevant considering the independence of milk-Zn concentrations on maternal diet (Moore et al., 1984; Krebs et al., 1995), and consistent with the fact that infants born to the lowest milk Zn-producing mothers (among other feeding factors) are more stunted than infants of women with higher milk Zn concentrations (Krebs et al., 1995; Umeta et al., 2000; Krebs and Hambidge, 2007).

## Author contributions

EN measured mitochondrial outcomes in mice at PND21 and 210, Zn, FMRP, and Shank3 levels, Zn in milk, run all western blots from rodents and human cells, analyzed the data, helped drafting, and reviewed the manuscript; CI measured mitochondrial outcomes isolated neurons and in mice at PND0-9 and PND21; GS measured ALP activity, proteins, and lactose levels in human milk and reviewed the manuscript; SW evaluated the *FMR1* expression in fibroblasts and *ZnT*s gene expression, and reviewed the manuscript; LG recruited the patients, collected the medical history, and the premutation milk samples; JS provided all milk samples from controls; FT determined the CGG repeats in fibroblasts and milk samples from gDNA extracted by Wong; CG conceptualized the work, wrote, and reviewed the manuscript.

## Funding

Support for this study was provided by the National Institutes of Health (ES020392, HD040661, and HD036071) and Simons Foundation (#271406). The funders had no role in study design, data collection and analysis, decision to publish, or preparation of the manuscript. The authors of this publication declare that they have no financial relationships relevant to this article to disclose.

### Conflict of interest statement

RH has received funding from Novartis, Roche/Genentech, Alcobra, and Neuren for treatment trials in fragile X syndrome, autism, and Down syndrome. She has also consulted with Novartis and Roche/Genentech regarding treatment for fragile X syndrome. The other authors declare that the research was conducted in the absence of any commercial or financial relationships that could be construed as a potential conflict of interest.

## References

[B1] Bañez-Coronel M. Porta S. Kagerbauer B. Mateu-Huertas E. Pantano L. Ferrer I.. (2012). A pathogenic mechanism in Huntington's disease involves small CAG-repeated RNAs with neurotoxic activity. PLoS Genet. 8:e1002481. 10.1371/journal.pgen.1002481 22383888 PMC3285580

[B2] Bardoni B. Mandel J. L. (2002). Advances in understanding of fragile X pathogenesis and FMRP function, and in identification of X linked mental retardation genes. Curr. Opin. Genet. Dev. 12, 284–293. 10.1016/S0959-437X(02)00300-3 12076671

[B3] Battistella G. Niederhauser J. Fornari E. Hippolyte L. Gronchi Perrin A. Lesca G.. (2013). Brain structure in asymptomatic FMR1 premutation carriers at risk for fragile X-associated tremor/ataxia syndrome. Neurobiol. Aging 34, 1700–1707. 10.1016/j.neurobiolaging.2012.12.001 23298734

[B4] Beach R. S. Gershwin M. E. Hurley L. S. (1982). Gestational zinc deprivation in mice: persistence of immunodeficiency for three generations. Science 218, 469–471. 10.1126/science.7123244 7123244

[B5] Belanger M. Magistretti P. J. (2009). The role of astroglia in neuroprotection. Dialogues Clin. Neurosci. 11, 281–295. 19877496 10.31887/DCNS.2009.11.3/mbelanger PMC3181926

[B6] Boldogh I. R. Pon L. A. (2006). Interactions of mitochondria with the actin cytoskeleton. Biochim. Biophys. Acta 1763, 450–462. 10.1016/j.bbamcr.2006.02.014 16624426

[B7] Brand M. D. (1990). The contribution of the leak of protons across the mitochondrial inner membrane to standard metabolic rate. J. Theor. Biol. 145, 267–286. 10.1016/S0022-5193(05)80131-6 2169556

[B8] Brody T. (1999). Nutritional Biochemistry. San Diego, CA: Academic Press.

[B9] Brown V. Jin P. Ceman S. Darnell J. C. O'Donnell W. T. Tenenbaum S. A.. (2001). Microarray identification of FMRP-associated brain mRNAs and altered mRNA translational profiles in fragile X syndrome. Cell 107, 477–487. 10.1016/S0092-8674(01)00568-2 11719188

[B10] Butterworth R. F. Heroux M. (1989). Effect of pyrithiamine treatment and subsequent thiamine rehabilitation on regional cerebral amino acids and thiamine-dependent enzymes. J. Neurochem. 52, 1079–1084. 10.1111/j.1471-4159.1989.tb01850.x 2564421

[B11] Castets M. Schaeffer C. Bechara E. Schenck A. Khandjian E. W. Luche S.. (2005). FMRP interferes with the Rac1 pathway and controls actin cytoskeleton dynamics in murine fibroblasts. Hum. Mol. Genet. 14, 835–844. 10.1093/hmg/ddi077 15703194

[B12] Chanda R. Owen E. C. Cramond B. (1951). The composition of human milk with special reference to the relation between phosphorus partition and phosphatase and to the partition of certain vitamins. Br. J. Nutr. 5, 228–242. 10.1079/BJN19510029 14886540

[B13] Chonchaiya W. Tardif T. Mai X. Xu L. Li M. Kaciroti N.. (2013). Developmental trends in auditory processing can provide early predictions of language acquisition in young infants. Dev. Sci. 16, 159–172. 10.1111/desc.12012 23432827 PMC3582039

[B14] Coburn S. P. Mahuren J. D. Pauly T. A. Ericson K. L. Townsend D. W. (1992). Alkaline phosphatase activity and pyridoxal phosphate concentrations in the milk of various species. J. Nutr. 122, 2348–2353. 1453218 10.1093/jn/122.12.2348

[B15] Darnell J. C. Richter J. D. (2012). Cytoplasmic RNA-binding proteins and the control of complex brain function. Cold Spring Harb. Perspect. Biol. 4:a012344. 10.1101/cshperspect.a012344 22723494 PMC3405866

[B16] Darnell J. C. Van Driesche S. J. Zhang C. Hung K. Y. Mele A. Fraser C. E.. (2011). FMRP stalls ribosomal translocation on mRNAs linked to synaptic function and autism. Cell 146, 247–261. 10.1016/j.cell.2011.06.013 21784246 PMC3232425

[B17] D'Hulst C. Heulens I. Brouwer J. R. Willemsen R. De Geest N. Reeve S. P.. (2009). Expression of the GABAergic system in animal models for fragile X syndrome and fragile X associated tremor/ataxia syndrome (FXTAS). Brain Res. 1253, 176–183. 10.1016/j.brainres.2008.11.075 19070606

[B18] Dugina V. Zwaenepoel I. Gabbiani G. Clement S. Chaponnier C. (2009). Beta and gamma-cytoplasmic actins display distinct distribution and functional diversity. J. Cell Sci. 122, 2980–2988. 10.1242/jcs.041970 19638415

[B19] Elfering S. L. Haynes V. L. Traaseth N. J. Ettl A. Giulivi C. (2004). Aspects, mechanism, and biological relevance of mitochondrial protein nitration sustained by mitochondrial nitric oxide synthase. Am. J. Physiol. Heart Circ. Physiol. 286, H22–H29. 10.1152/ajpheart.00766.2003 14527943

[B20] Farzin F. Perry H. Hessl D. Loesch D. Cohen J. Bacalman S.. (2006). Autism spectrum disorders and attention-deficit/hyperactivity disorder in boys with the fragile X premutation. J. Dev. Behav. Pediatr. 27, S137–S144. 10.1097/00004703-200604002-00012 16685180

[B21] Fransson G. B. Lonnerdal B. (1984). Iron, copper, zinc, calcium, and magnesium in human milk fat. Am. J. Clin. Nutr. 39, 185–189. 6695823 10.1093/ajcn/39.2.185

[B22] Fujisawa Y. Napoli E. Wong S. Song G. Yamaguchi R. Matsui T.. (2015). Impact of a novel homozygous mutation in nicotinamide nucleotide transhydrogenase on mitochondrial DNA integrity in a case of familial glucocorticoid deficiency. Proc. Natl. Acad. Sci. U.S.A. 3, 70–78. 10.1016/j.bbacli.2014.12.003 26309815 PMC4545511

[B23] Garcia-Nonell C. Ratera E. R. Harris S. Hessl D. Ono M. Y. Tartaglia N.. (2008). Secondary medical diagnosis in fragile X syndrome with and without autism spectrum disorder. Am. J. Med. Genet. A 146A, 1911–1916. 10.1002/ajmg.a.32290 18627038 PMC4097171

[B24] Grabrucker S. Jannetti L. Eckert M. Gaub S. Chhabra R. Pfaender S.. (2014). Zinc deficiency dysregulates the synaptic ProSAP/Shank scaffold and might contribute to autism spectrum disorders. Brain 137, 137–152. 10.1093/brain/awt303 24277719

[B25] Greco C. M. Berman R. F. Martin R. M. Tassone F. Schwartz P. H. Chang A.. (2006). Neuropathology of fragile X-associated tremor/ataxia syndrome (FXTAS). Brain 129, 243–255. 10.1093/brain/awh683 16332642

[B26] Hagerman R. Hagerman P. (2013). Advances in clinical and molecular understanding of the FMR1 premutation and fragile X-associated tremor/ataxia syndrome. Lancet Neurol. 12, 786–798. 10.1016/S1474-4422(13)70125-X 23867198 PMC3922535

[B27] Hagerman R. Hoem G. Hagerman P. (2010). Fragile X and autism: intertwined at the molecular level leading to targeted treatments. Mol. Autism 1:12. 10.1186/2040-2392-1-12 20858229 PMC2954865

[B28] Han K. Holder J. L. Jr. Schaaf C. P. Lu H. Chen H. Kang H.. (2013). SHANK3 overexpression causes manic-like behaviour with unique pharmacogenetic properties. Nature 503, 72–77. 10.1038/nature12630 24153177 PMC3923348

[B29] Hara Y. Yuk F. Puri R. Janssen W. G. Rapp P. R. Morrison J. H. (2014). Presynaptic mitochondrial morphology in monkey prefrontal cortex correlates with working memory and is improved with estrogen treatment. Proc. Natl. Acad. Sci. U.S.A. 111, 486–491. 10.1073/pnas.1311310110 24297907 PMC3890848

[B30] Harris S. W. Hessl D. Goodlin-Jones B. Ferranti J. Bacalman S. Barbato I.. (2008). Autism profiles of males with fragile X syndrome. Am. J. Ment. Retard. 113, 427–438. 10.1352/2008.113:427-438 19127654 PMC2629645

[B31] Hatch A. L. Gurel P. S. Higgs H. N. (2014). Novel roles for actin in mitochondrial fission. J. Cell Sci. 127, 4549–4560. 10.1242/jcs.153791 25217628 PMC4215709

[B32] Haynes V. Traaseth N. J. Elfering S. Fujisawa Y. Giulivi C. (2010). Nitration of specific tyrosines in FoF1 ATP synthase and activity loss in aging. Am. J. Physiol. Endocrinol. Metab. 298, E978–E987. 10.1152/ajpendo.00739.2009 20159857 PMC2867368

[B33] Higuchi R. Vevea J. D. Swayne T. C. Chojnowski R. Hill V. Boldogh I. R.. (2013). Actin dynamics affect mitochondrial quality control and aging in budding yeast. Curr. Biol. 23, 2417–2422. 10.1016/j.cub.2013.10.022 24268413 PMC3932488

[B34] Ho E. Courtemanche C. Ames B. N. (2003). Zinc deficiency induces oxidative DNA damage and increases p53 expression in human lung fibroblasts. J. Nutr. 133, 2543–2548. 12888634 10.1093/jn/133.8.2543

[B35] Huang L. Gitschier J. (1997). A novel gene involved in zinc transport is deficient in the lethal milk mouse. Nat. Genet. 17, 292–297. 10.1038/ng1197-292 9354792

[B36] Hunsaker M. R. Arque G. Berman R. F. Willemsen R. Hukema R. K. (2012). Mouse models of the fragile x premutation and the fragile X associated tremor/ataxia syndrome. Results Probl. Cell Differ. 54, 255–269. 10.1007/978-3-642-21649-7_14 22009357 PMC4313770

[B37] Hunsaker M. R. von Leden R. E. Ta B. T. Goodrich-Hunsaker N. J. Arque G. Kim K.. (2011). Motor deficits on a ladder rung task in male and female adolescent and adult CGG knock-in mice. Behav. Brain Res. 222, 117–121. 10.1016/j.bbr.2011.03.039 21440572 PMC3095688

[B38] Hunsaker M. R. Wenzel H. J. Willemsen R. Berman R. F. (2009). Progressive spatial processing deficits in a mouse model of the fragile X premutation. Behav. Neurosci. 123, 1315–1324. 10.1037/a0017616 20001115 PMC3410547

[B39] Isaya G. Kalousek F. Fenton W. A. Rosenberg L. E. (1991). Cleavage of precursors by the mitochondrial processing peptidase requires a compatible mature protein or an intermediate octapeptide. J. Cell Biol. 113, 65–76. 10.1083/jcb.113.1.65 1672532 PMC2288917

[B40] Iwata-Ichikawa E. Kondo Y. Miyazaki I. Asanuma M. Ogawa N. (1999). Glial cells protect neurons against oxidative stress via transcriptional up-regulation of the glutathione synthesis. J. Neurochem. 72, 2334–2344. 10.1046/j.1471-4159.1999.0722334.x 10349842

[B41] Jagoda C. A. Rillema J. A. (1991). Temporal effect of prolactin on the activities of lactose synthetase, alpha-lactalbumin, and galactosyl transferase in mouse mammary gland explants. Proc. Soc. Exp. Biol. Med. 197, 431–434. 10.3181/00379727-197-43278 1908098

[B42] Jekabsons M. B. Nicholls D. G. (2004). *In situ* respiration and bioenergetic status of mitochondria in primary cerebellar granule neuronal cultures exposed continuously to glutamate. J. Biol. Chem. 279, 32989–33000. 10.1074/jbc.M401540200 15166243

[B43] Kaplan E. S. Cao Z. Hulsizer S. Tassone F. Berman R. F. Hagerman P. J.. (2012). Early mitochondrial abnormalities in hippocampal neurons cultured from Fmr1 pre-mutation mouse model. J. Neurochem. 123, 613–621. 10.1111/j.1471-4159.2012.07936.x 22924671 PMC3564636

[B44] Kelleher S. L. Velasquez V. Croxford T. P. McCormick N. H. Lopez V. MacDavid J. (2012). Mapping the zinc-transporting system in mammary cells: molecular analysis reveals a phenotype-dependent zinc-transporting network during lactation. J. Cell. Physiol. 227, 1761–1770. 10.1002/jcp.22900 21702047 PMC3207005

[B45] Kenneson A. Zhang F. Hagedorn C. H. Warren S. T. (2001). Reduced FMRP and increased FMR1 transcription is proportionally associated with CGG repeat number in intermediate-length and premutation carriers. Hum. Mol. Genet. 10, 1449–1454. 10.1093/hmg/10.14.1449 11448936

[B46] Kim S. Y. Hashimoto R. Tassone F. Simon T. J. Rivera S. M. (2013). Altered neural activity of magnitude estimation processing in adults with the fragile X premutation. J. Psychiatr. Res. 47, 1909–1916. 10.1016/j.jpsychires.2013.08.014 24045061 PMC3880247

[B47] Knapka J. J. Smith K. P. Judge F. J. (1974). Effect of open and closed formula rations on the performance of three strains of laboratory mice. Lab. Anim. Sci. 24, 480–487. 4365336

[B48] Kogan C. S. Turk J. Hagerman R. J. Cornish K. M. (2008). Impact of the Fragile X mental retardation 1 (FMR1) gene premutation on neuropsychiatric functioning in adult males without fragile X-associated Tremor/Ataxia syndrome: a controlled study. Am. J. Med. Genet. B Neuropsychiatr. Genet. 147B, 859–872. 10.1002/ajmg.b.30685 18165971

[B49] Korobova F. Ramabhadran V. Higgs H. N. (2013). An actin-dependent step in mitochondrial fission mediated by the ER-associated formin INF2. Science 339, 464–467. 10.1126/science.1228360 23349293 PMC3843506

[B50] Kouser M. Speed H. E. Dewey C. M. Reimers J. M. Widman A. J. Gupta N.. (2013). Loss of predominant Shank3 isoforms results in hippocampus-dependent impairments in behavior and synaptic transmission. J. Neurosci. 33, 18448–18468. 10.1523/JNEUROSCI.3017-13.2013 24259569 PMC3834052

[B51] Krebs N. F. Hambidge K. M. (2007). Complementary feeding: clinically relevant factors affecting timing and composition. Am. J. Clin. Nutr. 85, 639S–645S. 17284770 10.1093/ajcn/85.2.639S

[B52] Krebs N. F. Reidinger C. J. Hartley S. Robertson A. D. Hambidge K. M. (1995). Zinc supplementation during lactation: effects on maternal status and milk zinc concentrations. Am. J. Clin. Nutr. 61, 1030–1036. 7733024 10.1093/ajcn/61.4.1030

[B53] Kusano H. Shimizu S. Koya R. C. Fujita H. Kamada S. Kuzumaki N.. (2000). Human gelsolin prevents apoptosis by inhibiting apoptotic mitochondrial changes via closing VDAC. Oncogene 19, 4807–4814. 10.1038/sj.onc.1203868 11039896

[B54] Li J. Li Q. Xie C. Zhou H. Wang Y. Zhang N.. (2004). Beta-actin is required for mitochondria clustering and ROS generation in TNF-induced, caspase-independent cell death. J. Cell Sci. 117, 4673–4680. 10.1242/jcs.01339 15371523

[B55] Li S. Xu S. Roelofs B. A. Boyman L. Lederer W. J. Sesaki H.. (2015). Transient assembly of F-actin on the outer mitochondrial membrane contributes to mitochondrial fission. J. Cell Biol. 208, 109–123. 10.1083/jcb.201404050 25547155 PMC4284235

[B56] Ludwig A. L. Espinal G. M. Pretto D. I. Jamal A. L. Arque G. Tassone F.. (2014). CNS expression of murine fragile X protein (FMRP) as a function of CGG-repeat size. Hum. Mol. Genet. 23, 3228–3238. 10.1093/hmg/ddu032 24463622 PMC4030777

[B57] Luecke R. W. Fraker P. J. (1979). The effect of varying dietary zinc levels on growth and antibody-mediated response in two strains of mice. J. Nutr. 109, 1373–1376. 379282 10.1093/jn/109.8.1373

[B58] McCormick N. H. Kelleher S. L. (2012). ZnT4 provides zinc to zinc-dependent proteins in the trans-Golgi network critical for cell function and Zn export in mammary epithelial cells. Am. J. Physiol. Cell Physiol. 303, C291–C297. 10.1152/ajpcell.00443.2011 22621784 PMC3423030

[B59] Menalled L. El-Khodor B. F. Patry M. Suarez-Farinas M. Orenstein S. J. Zahasky B.. (2009). Systematic behavioral evaluation of Huntington's disease transgenic and knock-in mouse models. Neurobiol. Dis. 35, 319–336. 10.1016/j.nbd.2009.05.007 19464370 PMC2728344

[B60] Mohammad M. A. Hadsell D. L. Haymond M. W. (2012). Gene regulation of UDP-galactose synthesis and transport: potential rate-limiting processes in initiation of milk production in humans. Am. J. Physiol. Endocrinol. Metab. 303, E365–E376. 10.1152/ajpendo.00175.2012 22649065 PMC3423122

[B61] Moore M. E. Moran J. R. Greene H. L. (1984). Zinc supplementation in lactating women: evidence for mammary control of zinc secretion. J. Pediatr. 105, 600–602. 10.1016/S0022-3476(84)80430-8 6548259

[B62] Nagra S. A. (1989). Longitudinal study in biochemical composition of human milk during first year of lactation. J. Trop. Pediatr. 35, 126–128. 10.1093/tropej/35.3.126 2754770

[B63] Napoli E. Hung C. Wong S. Giulivi C. (2013). Toxicity of the flame-retardant BDE-49 on brain mitochondria and neuronal progenitor striatal cells enhanced by a PTEN-deficient background. Toxicol. Sci. 132, 196–210. 10.1093/toxsci/kfs339 23288049 PMC3693513

[B64] Napoli E. Ross-Inta C. Wong S. Hung C. Fujisawa Y. Sakaguchi D.. (2012). Mitochondrial dysfunction in Pten haplo-insufficient mice with social deficits and repetitive behavior: interplay between Pten and p53. PLoS ONE 7:e42504. 10.1371/journal.pone.0042504 22900024 PMC3416855

[B65] Napoli E. Ross-Inta C. Wong S. Omanska-Klusek A. Barrow C. Iwahashi C.. (2011). Altered zinc transport disrupts mitochondrial protein processing/import in fragile X-associated tremor/ataxia syndrome. Hum. Mol. Genet. 20, 3079–3092. 10.1093/hmg/ddr211 21558427 PMC3131047

[B66] Navarro D. Zwingmann C. Butterworth R. F. (2008). Region-selective alterations of glucose oxidation and amino acid synthesis in the thiamine-deficient rat brain: a re-evaluation using 1H/13C nuclear magnetic resonance spectroscopy. J. Neurochem. 106, 603–612. 10.1111/j.1471-4159.2008.05410.x 18410518

[B67] Nguyen L. S. Lepleux M. Makhlouf M. Martin C. Fregeac J. Siquier-Pernet K.. (2016). Profiling olfactory stem cells from living patients identifies miRNAs relevant for autism pathophysiology. Mol. Autism 7, 1. 10.1186/s13229-015-0064-6 26753090 PMC4705753

[B68] Nicholls D. G. (2008). Oxidative stress and energy crises in neuronal dysfunction. Ann. N.Y. Acad. Sci. 1147, 53–60. 10.1196/annals.1427.002 19076430

[B69] Nolze A. Schneider J. Keil R. Lederer M. Huttelmaier S. Kessels M. M.. (2013). FMRP regulates actin filament organization via the armadillo protein p0071. RNA 19, 1483–1496. 10.1261/rna.037945.112 24062571 PMC3851716

[B70] Oh S. Y. He F. Krans A. Frazer M. Taylor J. P. Paulson H. L.. (2015). RAN translation at CGG repeats induces ubiquitin proteasome system impairment in models of fragile X-associated tremor ataxia syndrome. Hum. Mol. Genet. 24, 4317–4326. 10.1093/hmg/ddv165 25954027 PMC4492395

[B71] Picard M. McEwen B. S. (2014). Mitochondria impact brain function and cognition. Proc. Natl. Acad. Sci. U.S.A. 111, 7–8. 10.1073/pnas.1321881111 24367081 PMC3890847

[B72] Pieretti M. Zhang F. P. Fu Y. H. Warren S. T. Oostra B. A. Caskey C. T.. (1991). Absence of expression of the FMR-1 gene in fragile X syndrome. Cell 66, 817–822. 10.1016/0092-8674(91)90125-I 1878973

[B73] Rendon A. Masmoudi A. (1985). Purification of non-synaptic and synaptic mitochondria and plasma membranes from rat brain by a rapid Percoll gradient procedure. J. Neurosci. Methods 14, 41–51. 10.1016/0165-0270(85)90113-X 2993759

[B74] Ross-Inta C. Omanska-Klusek A. Wong S. Barrow C. Garcia-Arocena D. Iwahashi C.. (2010). Evidence of mitochondrial dysfunction in fragile X-associated tremor/ataxia syndrome. Biochem. J. 429, 545–552. 10.1042/BJ20091960 20513237 PMC4011071

[B75] Russo A. J. Devito R. (2011). Analysis of copper and zinc plasma concentration and the efficacy of zinc therapy in individuals with asperger's syndrome, pervasive developmental disorder not otherwise specified (PDD-NOS) and autism. Biomark. Insights 6, 127–133. 10.4137/BMI.S7286 22174567 PMC3235993

[B76] Sawashita J. Takeda A. Okada S. (1997). Change of zinc distribution in rat brain with increasing age. Brain Res. Dev. Brain Res. 102, 295–298. 10.1016/S0165-3806(97)00107-7 9352113

[B77] Selkoe D. J. (2002). Alzheimer's disease is a synaptic failure. Science 298, 789–791. 10.1126/science.1074069 12399581

[B78] Sellier C. Freyermuth F. Tabet R. Tran T. He F. Ruffenach F.. (2013). Sequestration of DROSHA and DGCR8 by expanded CGG RNA repeats alters microRNA processing in fragile X-associated tremor/ataxia syndrome. Cell Rep. 3, 869–880. 10.1016/j.celrep.2013.02.004 23478018 PMC3639429

[B79] Sellier C. Rau F. Liu Y. Tassone F. Hukema R. K. Gattoni R.. (2010). Sam68 sequestration and partial loss of function are associated with splicing alterations in FXTAS patients. EMBO J. 29, 1248–1261. 10.1038/emboj.2010.21 20186122 PMC2857464

[B80] Stewart R. A. Platou E. Kelly V. J. (1958). The alkaline phosphatase content of human milk. J. Biol. Chem. 232, 777–784. 13549462

[B81] Sutcliffe J. S. Nelson D. L. Zhang F. Pieretti M. Caskey C. T. Saxe D.. (1992). DNA methylation represses FMR-1 transcription in fragile X syndrome. Hum. Mol. Genet. 1, 397–400. 10.1093/hmg/1.6.397 1301913

[B82] Tassone F. Beilina A. Carosi C. Albertosi S. Bagni C. Li L.. (2007). Elevated FMR1 mRNA in premutation carriers is due to increased transcription. RNA 13, 555–562. 10.1261/rna.280807 17283214 PMC1831862

[B83] Tassone F. Greco C. M. Hunsaker M. R. Seritan A. L. Berman R. F. Gane L. W.. (2012). Neuropathological, clinical and molecular pathology in female fragile X premutation carriers with and without FXTAS. Genes Brain Behav. 11, 577–585. 10.1111/j.1601-183X.2012.00779.x 22463693 PMC3965773

[B84] Tassone F. Hagerman P. J. (2003). Expression of the FMR1 gene. Cytogenet. Genome Res. 100, 124–128. 10.1159/000072846 14526172

[B85] Tassone F. Hagerman R. J. Loesch D. Z. Lachiewicz A. Taylor A. K. Hagerman P. J. (2000a). Fragile X males with unmethylated, full mutation trinucleotide repeat expansions have elevated levels of *FMR1* messenger RNA. Am. J. Med. Genet. 94, 232–236. 10.1002/1096-8628(20000918)94:3<232::AID-AJMG9>3.0.CO;2-H 10995510

[B86] Tassone F. Hagerman R. J. Taylor A. K. Gane L. W. Godfrey T. E. Hagerman P. J. (2000b). Elevated levels of *FMR1* mRNA in carrier males: a new mechanism of involvement in the fragile-X syndrome. Am. J. Hum. Genet. 66, 6–15. 10.1086/302720 10631132 PMC1288349

[B87] Tassone F. Hagerman R. J. Taylor A. K. Mills J. B. Harris S. W. Gane L. W.. (2000c). Clinical involvement and protein expression in individuals with the *FMR1* premutation. Am. J. Med. Genet. 91, 144–152. 10.1002/(SICI)1096-8628(20000313)91:2<144::AID-AJMG14>3.0.CO;2-V 10748416

[B88] Tassone F. Iwahashi C. Hagerman P. J. (2004). FMR1 RNA within the intranuclear inclusions of fragile X-associated tremor/ataxia syndrome (FXTAS). RNA Biol. 1, 103–105. 10.4161/rna.1.2.1035 17179750

[B89] Todd P. K. Oh S. Y. Krans A. He F. Sellier C. Frazer M.. (2013). CGG repeat-associated translation mediates neurodegeneration in fragile X tremor ataxia syndrome. Neuron 78, 440–455. 10.1016/j.neuron.2013.03.026 23602499 PMC3831531

[B90] Tokatlidis K. Vial S. Luciano P. Vergnolle M. Clémence S. (2000). Membrane protein import in yeast mitochondria. Biochem. Soc. Trans. 28, 495–499. 10.1042/bst0280495 10961947

[B91] Tudehope D. I. (2013). Human milk and the nutritional needs of preterm infants. J. Pediatr. 162, S17–S25. 10.1016/j.jpeds.2012.11.049 23445843

[B92] Umeta M. West C. E. Haidar J. Deurenberg P. Hautvast J. G. (2000). Zinc supplementation and stunted infants in Ethiopia: a randomised controlled trial. Lancet 355, 2021–2026. 10.1016/S0140-6736(00)02348-5 10885352

[B93] Van Dam D. Errijgers V. Kooy R. F. Willemsen R. Mientjes E. Oostra B. A.. (2005). Cognitive decline, neuromotor and behavioural disturbances in a mouse model for fragile-X-associated tremor/ataxia syndrome (FXTAS). Behav. Brain Res. 162, 233–239. 10.1016/j.bbr.2005.03.007 15876460

[B94] Wang X. Xu Q. Bey A. L. Lee Y. Jiang Y. H. (2014). Transcriptional and functional complexity of Shank3 provides a molecular framework to understand the phenotypic heterogeneity of SHANK3 causing autism and Shank3 mutant mice. Mol. Autism 5, 30. 10.1186/2040-2392-5-30 25071925 PMC4113141

[B95] Weiler I. J. Irwin S. A. Klintsova A. Y. Spencer C. M. Brazelton A. D. Miyashiro K.. (1997). Fragile X mental retardation protein is translated near synapses in response to neurotransmitter activation. Proc. Natl. Acad. Sci. U.S.A. 94, 5395–5400. 10.1073/pnas.94.10.5395 9144248 PMC24689

[B96] Wenzel H. J. Hunsaker M. R. Greco C. M. Willemsen R. Berman R. F. (2010). Ubiquitin-positive intranuclear inclusions in neuronal and glial cells in a mouse model of the fragile X premutation. Brain Res. 1318, 155–166. 10.1016/j.brainres.2009.12.077 20051238 PMC3086812

[B97] Winarni T. I. Chonchaiya W. Sumekar T. A. Ashwood P. Morales G. M. Tassone F.. (2012). Immune-mediated disorders among women carriers of fragile X premutation alleles. Am. J. Med. Genet. A 158A, 2473–2481. 10.1002/ajmg.a.35569 22903889 PMC4105154

[B98] Wong L. M. Goodrich-Hunsaker N. J. McLennan Y. Tassone F. Harvey D. Rivera S. M.. (2012). Young adult male carriers of the fragile X premutation exhibit genetically modulated impairments in visuospatial tasks controlled for psychomotor speed. J. Neurodev. Disord. 4:26. 10.1186/1866-1955-4-26 23148490 PMC3506571

[B99] Worth G. K. Retallack R. W. Gutteridge D. H. Jefferies M. Kent J. Smith M. (1981). Serum and milk alkaline phosphatase in human lactation. Clin. Chim. Acta 115, 171–177. 10.1016/0009-8981(81)90073-5 7197203

[B100] Xu X. Forbes J. G. Colombini M. (2001). Actin modulates the gating of Neurospora crassa VDAC. J. Membr. Biol. 180, 73–81. 10.1007/s002320010060 11284205

[B101] Yadava N. Nicholls D. G. (2007). Spare respiratory capacity rather than oxidative stress regulates glutamate excitotoxicity after partial respiratory inhibition of mitochondrial complex I with rotenone. J. Neurosci. 27, 7310–7317. 10.1523/JNEUROSCI.0212-07.2007 17611283 PMC6794596

[B102] Yasuda H. Yoshida K. Yasuda Y. Tsutsui T. (2011). Infantile zinc deficiency: association with autism spectrum disorders. Sci. Rep. 1:129. 10.1038/srep00129 22355646 PMC3216610

[B103] Zalewski P. Truong-Tran A. Lincoln S. Ward D. Shankar A. Coyle P.. (2006). Use of a zinc fluorophore to measure labile pools of zinc in body fluids and cell-conditioned media. BioTechniques 40, 509–520. 10.2144/06404RR02 16629398

[B104] Zalfa F. Giorgi M. Primerano B. Moro A. Di Penta A. Reis S.. (2003). The fragile X syndrome protein FMRP associates with BC1 RNA and regulates the translation of specific mRNAs at synapses. Cell 112, 317–327. 10.1016/S0092-8674(03)00079-5 12581522

[B105] Zingerevich C. Greiss-Hess L. Lemons-Chitwood K. Harris S. W. Hessl D. Cook K.. (2009). Motor abilities of children diagnosed with fragile X syndrome with and without autism. J. Intellect. Disabil. Res. 53, 11–18. 10.1111/j.1365-2788.2008.01107.x 18771512 PMC2614297

[B106] Zongaro S. Hukema R. D'Antoni S. Davidovic L. Barbry P. Catania M. V.. (2013). The 3′ UTR of FMR1 mRNA is a target of miR-101, miR-129-5p and miR-221: implications for the molecular pathology of FXTAS at the synapse. Hum. Mol. Genet. 22, 1971–1982. 10.1093/hmg/ddt044 23390134

