# Supplementary material for: Premutation in the Fragile X Mental Retardation 1 (FMR1) Gene Affects Maternal Zn-milk and Perinatal Brain Bioenergetics and Scaffolding
Source: Front Neurosci. 2016 Apr 19;10:159. doi: 10.3389/fnins.2016.00159 (PMC4835505; doi:10.3389/fnins.2016.00159)
Supplement: Supplementary file 1 [file Image1.PDF]

*Supplementary Material*

# Premutation in the Fragile X Mental Retardation 1 (FMR1) Gene Affects Maternal Zn-milk and Perinatal Brain Bioenergetics and Scaffolding

**Eleonora Napoli, Catherine Ross-Inta, Gyu Song, Sarah Wong, Randi Hagerman, Louise W. Gane, Jennifer T. Smilowitz, Flora Tassone, and Cecilia Giulivi***

*** Corresponding Author: cgiulivi@ucdavis.edu**

## Supplementary Figures

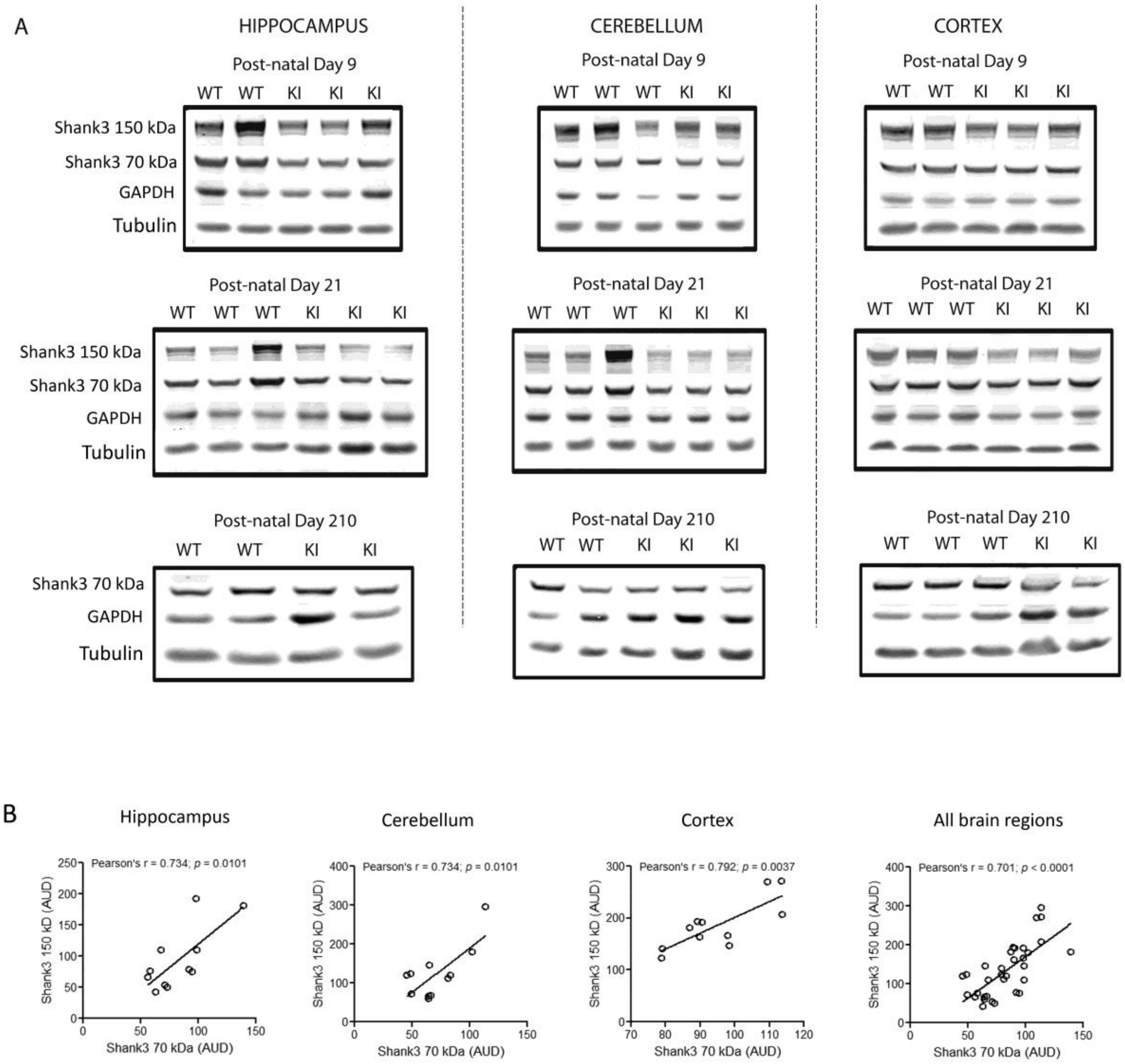


C

| Brain area/Age | Genotype | Shank3/Tubulin[a] (% WT) | Shank3/Tubulin[b] (% WT) | Shank3/GAPDH[c] (% WT) | ANOVA summary F (P value) |
|---|---|---|---|---|---|
| Hippocampus/PND9 | WT | 100 ± 5 | 100 ± 20 | 100 ± 34 | |
| | KI | 58 ± 4 | 46 ± 15 | 49 ± 5 | 1.776 (0.238) |
| Hippocampus/PND21 | WT | 100 ± 56 | 100 ± 63 | 100 ± 76 | |
| | KI | 38 ± 19 | 24 ± 3 | 37 ± 15 | 0.877 (0.457) |
| Cerebellum/PND9 | WT | 100 ± 7 | 100 ± 14 | 100 ± 25 | |
| | KI | 58 ± 10 | 61 ± 14 | 84 ± 15 | 2.978 (0.107) |
| Cerebellum/PND21 | WT | 100 ± 14 | 100 ± 56 | 100 ± 68 | |
| | KI | 45 ± 7 | 40 ± 2 | 40 ± 5 | 1.024 (0.407) |
| Cortex/PND9 | WT | 100 ± 18 | 100 ± 10 | 100 ± 9 | |
| | KI | 101 ± 50 | 74 ± 10 | 66 ± 4 | 1.260 (0.335) |
| Cortex/PND21 | WT | 100 ± 33 | 100 ± 8 | 100 ± 13 | |
| | KI | 79 ± 43 | 59 ± 8 | 79 ± 1 | 0.709 (0.520) |

**Supplementary Figure 1. (A)** Representative Western blots for Shank3 protein expression in hippocampus, cerebellum and cortex of WT and KI mice at PND9, 21 and 210. Samples were concentrated and partly delipidated by using acetone precipitation as described in the Methods section. Two Shank3 isoforms (150 and 70 kDa) are shown at PND9 and PND21. Due to the age-dependent decrease in Shank3 expression and the acetone precipitation, the band at PND210 was not reliably detected. **(B)** Correlation between protein expression levels of the 150 and 70 kDa isoforms of Shank3 in hippocampus, cerebellum and cortex. **(C)** Shank3 expression levels in brain regions from KI mice relative to WT in (***a***) samples normalized to tubulin, (***b***) acetone-treated samples normalized to tubulin, and (***c***) acetone-treated samples normalized to GAPDH. Shank3 was expressed as % of WT at each time-point and reported as mean ± SD. No statistically significant difference (*per* 1-way ANOVA analysis) was recorded in the expression levels of Shank3 in KI relative to WT across the 3 protocols utilized.
